# Lifestyle coaching is feasible in fatigued brain tumor patients: A phase I/feasibility, multi-center, mixed-methods randomized controlled trial

**DOI:** 10.1093/nop/npac086

**Published:** 2022-10-14

**Authors:** Alasdair G Rooney, William Hewins, Amie Walker, Mairi Mackinnon, Lisa Withington, Sara Robson, Claire Torrens, Lisa E M Hopcroft, Antony Clark, Garry Anderson, Helen Bulbeck, Joanna Dunlop, Michelle Welsh, Aimee Dyson, Julie Emerson, Carol Cochrane, Robert Hill, Jade Carruthers, Julia Day, David Gillespie, Christopher Hewitt, Emanuela Molinari, Mary Wells, Catherine McBain, Anthony J Chalmers, Robin Grant

**Affiliations:** Centre for Clinical Brain Sciences, University of Edinburgh, UK; The Robert Fergusson Unit, Royal Edinburgh Hospital, Edinburgh, UK; Department of Clinical Neurosciences, Edinburgh Centre for Neuro-Oncology, Royal Infirmary of Edinburgh, Edinburgh, UK; Centre for Clinical Brain Sciences, University of Edinburgh, UK; Department of Clinical Neurosciences, Edinburgh Centre for Neuro-Oncology, Royal Infirmary of Edinburgh, Edinburgh, UK; Centre for Clinical Brain Sciences, University of Edinburgh, UK; Neuro-Oncology, Beatson West of Scotland Cancer Centre, Glasgow, UK; Neuro-Oncology, Beatson West of Scotland Cancer Centre, Glasgow, UK; Clinical Oncology, The Christie NHS Foundation Trust, Manchester, UK; Clinical Oncology, The Christie NHS Foundation Trust, Manchester, UK; Nursing, Midwifery, and Allied Health Professions Research Unit, University of Stirling, Stirling, UK; Scottish Clinical Trials Research Unit (SCTRU), Public Health Scotland, Edinburgh, UK; Scottish Clinical Trials Research Unit (SCTRU), Public Health Scotland, Edinburgh, UK; V3 Health, Linlithgow, UK; brainstrust, the Brain Cancer People, UK; Scottish Clinical Trials Research Unit (SCTRU), Public Health Scotland, Edinburgh, UK; Community Rehabilitation and Brain Injury Service, Livingston, UK; Department of Clinical Neurosciences, Edinburgh Centre for Neuro-Oncology, Royal Infirmary of Edinburgh, Edinburgh, UK; Scottish Clinical Trials Research Unit (SCTRU), Public Health Scotland, Edinburgh, UK; Department of Surgery and Cancer, Imperial College Healthcare NHS Trust, London, UK; Clinical Oncology, The Christie NHS Foundation Trust, Manchester, UK; Clinical Oncology, The Christie NHS Foundation Trust, Manchester, UK; brainstrust, the Brain Cancer People, UK; Scottish Clinical Trials Research Unit (SCTRU), Public Health Scotland, Edinburgh, UK; Scottish Clinical Trials Research Unit (SCTRU), Public Health Scotland, Edinburgh, UK; Scottish Clinical Trials Research Unit (SCTRU), Public Health Scotland, Edinburgh, UK; Community Rehabilitation and Brain Injury Service, Livingston, UK; Department of Clinical Neurosciences, Edinburgh Centre for Neuro-Oncology, Royal Infirmary of Edinburgh, Edinburgh, UK; Centre for Clinical Brain Sciences, University of Edinburgh, UK; Department of Clinical Neurosciences, Edinburgh Centre for Neuro-Oncology, Royal Infirmary of Edinburgh, Edinburgh, UK; Clinical Health Psychology, Astley-Ainslie Hospital, Edinburgh, UK; Institute of Neurosciences, Queen Elizabeth University Hospital, Glasgow, UK; Scottish Clinical Trials Research Unit (SCTRU), Public Health Scotland, Edinburgh, UK; Imperial College Healthcare NHS Trust, London, UK; Clinical Oncology, The Christie NHS Foundation Trust, Manchester, UK; Neuro-Oncology, Beatson West of Scotland Cancer Centre, Glasgow, UK; Institute of Neurosciences, Queen Elizabeth University Hospital, Glasgow, UK; Centre for Clinical Brain Sciences, University of Edinburgh, UK; Department of Clinical Neurosciences, Edinburgh Centre for Neuro-Oncology, Royal Infirmary of Edinburgh, Edinburgh, UK

**Keywords:** brain tumor, coaching, fatigue, lifestyle, RCT

## Abstract

**Background:**

There are no effective treatments for brain tumor-related fatigue. We studied the feasibility of two novel lifestyle coaching interventions in fatigued brain tumor patients.

**Methods:**

This phase I/feasibility multi-center RCT recruited patients with a clinically stable primary brain tumor and significant fatigue (mean Brief Fatigue Inventory [BFI] score ≥ 4/10). Participants were randomized in a 1–1–1 allocation ratio to: Control (usual care); Health Coaching (“HC”, an eight-week program targeting lifestyle behaviors); or HC plus Activation Coaching (“HC + AC”, further targeting self-efficacy). The primary outcome was feasibility of recruitment and retention. Secondary outcomes were intervention acceptability, which was evaluated via qualitative interview, and safety. Exploratory quantitative outcomes were measured at baseline (T0), post-interventions (T1, 10 weeks), and endpoint (T2, 16 weeks).

**Results:**

*n* = 46 fatigued brain tumor patients (T0 BFI mean = 6.8/10) were recruited and 34 were retained to endpoint, establishing feasibility. Engagement with interventions was sustained over time. Qualitative interviews (*n* = 21) suggested that coaching interventions were broadly acceptable, although mediated by participant outlook and prior lifestyle. Coaching led to significant improvements in fatigue (improvement in BFI versus control at T1: HC=2.2 points [95% CI 0.6, 3.8], HC + AC = 1.8 [0.1, 3.4], Cohen’s *d* [HC] = 1.9; improvement in FACIT-Fatigue: HC = 4.8 points [−3.7, 13.3]; HC + AC = 12 [3.5, 20.5], *d* [HC and AC] = 0.9). Coaching also improved depressive and mental health outcomes. Modeling suggested a potential limiting effect of higher baseline depressive symptoms.

**Conclusions:**

Lifestyle coaching interventions are feasible to deliver to fatigued brain tumor patients. They were manageable, acceptable, and safe, with preliminary evidence of benefit on fatigue and mental health outcomes. Larger trials of efficacy are justified.

Brain tumor-related fatigue is a common and pervasive problem. Frequency estimates for fatigue range from 39 to 96%^1,2^ seemingly irrespective of brain tumor grade,^3^ histology,^4^ or clinical time-point,^5^ and often comorbid with cognitive impairment or depressive symptoms.^2,6^ The profound impact of fatigue^4,7^ is widely recognized. For example, The Brain Tumour Charity highlights its devastating impact on a person’s capacity to cope with daily life,^8^ while the UK James Lind Alliance considers the management of fatigue to be a top research priority for the neuro-oncology community.^9^

There is a lack of evidence to underpin effective management of brain tumor-related fatigue,^10^ despite several psychostimulant drug trials over the past decade. Trials of Methylphenidate and Armodafinil included non-fatigued patients,^11–13^ while studies of Modafinil, Dexamphetamine, and Armodafinil recruited only highly fatigued patients but found no evidence of benefit over placebo on their primary outcome of fatigue.^14–16^

Non-pharmacological treatments are a proposed alternative to medication for many cancer-related symptoms.^17–19^ Some of these treatments incorporate more complex “lifestyle coaching” approaches that aim to reinforce healthy behavior,^20^ social activity,^21^ stress reduction,^22^ exercise,^23^ and/or better dietary habits.^24^ Lifestyle coaching interventions have shown promise in alleviating fatigue in patients with cancers arising out-with the CNS.^25^ Within neuro-oncology it was established recently that exercise^23^ and yoga^26^ are feasible to deliver to non-fatigued patients. However, lifestyle coaching interventions have not been studied in highly fatigued patients. These patients are often quite impaired, and it is far from clear whether they would find such interventions feasible and acceptable.

We therefore developed BT-LIFE (***B***rain ***T***umors, ***L***ifestyle ***I***nterventions, and ***F***atigue ***E***valuation), a pilot (Phase I/feasibility) randomized controlled trial of two lifestyle coaching interventions for clinically significant brain tumor-related fatigue. The first intervention, “Health Coaching”, promoted a healthy lifestyle. The second, “Activation Coaching”, promoted self-efficacy. Both interventions are described in detail below. Our primary aim was to determine the feasibility of recruiting and retaining brain tumor patients with moderate or severe fatigue to HC and AC interventions. Secondary aims were to determine interventional acceptability, engagement, and safety; and to gather exploratory outcome data to inform a larger trial.

## Methods

### Trial Design

BT-LIFE was a Phase I/feasibility, multi-center, mixed-methods, three-arm randomized controlled trial with 1:1:1 allocation stratifying by center (ISRCTN17883425, Supplementary Figure S1). The study was coordinated by the Scottish Clinical Trials Research Unit. The final study protocol is presented in Supplementary Methods.

### Participants

Eligible participants were: aged 18+; diagnosed with a primary brain tumor of any grade or histological subtype; ≥ 3 months after completion of primary treatment (any combination of surgery, radiotherapy and/or chemotherapy) with no evidence of disease progression; and clinically significantly fatigued (mean Brief Fatigue Inventory [BFI] score of at least 4/10).^27^ Exclusion criteria were: low fatigue (mean BFI < 4/10); clinical concern about disease progression; clinically adjudged severe cognitive, language, or visual impairment; or inability to give informed consent.

Participants were recruited from neuro-oncology outpatient clinics in three UK centers (Edinburgh, Glasgow, and Manchester). Patients describing fatigue in their usual clinical appointment and who showed interest in the trial were screened for eligibility. WHO tumor grade was based on WHO 2016 diagnostic criteria. Baseline and follow-up trial outcome data were gathered face-to-face. These outcome assessments usually occurred in clinical settings with reimbursement of travel costs. Rarely (< 5% of cases) other locations were used such as the participant’s home.

### Interventions

#### Control.

—Control arm participants received the Brain Tumour Charity’s information leaflet about fatigue and “Usual Care”. The information leaflet included written advice on managing fatigue. In our centers Usual Care included ongoing access to the neuro-oncology clinical team with routine scheduled follow-up appointments.

#### Health Coaching (HC).

—Participants in this arm received the information leaflet plus Health Coaching (hereafter HC). HC was a multimodal lifestyle coaching intervention which promoted incremental patient-led improvements in: fluid intake (reducing caffeine and alcohol and drinking more water); sleep (promoting rest); diet (encouraging healthy eating); exercise and movement (increasing number of steps per day); and reducing stress. Our HC model was developed in private practice by an accredited personal trainer. This individual (GA) delivered HC to patients recruited in Edinburgh and Glasgow. In Manchester HC was delivered by an NHS physiotherapist (AD) working to the same format.

Participants were offered eight weekly HC sessions. In this pilot study none of the sessions were formally manualized. However, efforts were made to standardize them between sites and patients. The initial session was a standardized 45-min face-to-face assessment with the Health Coach (Supplementary Methods). Each participant was provided with an infographic reminder card (Supplementary Figure S2), a wearable step counter (Omnicron), and a daily home diary (Supplementary Figure S3). Up to seven 30-min follow-up sessions were then delivered weekly by telephone or in person, informed by the diary and tailored to individual goals. These follow-up sessions followed a set structure (Supplementary Methods) with their content reflecting the goals and progress of individual patients.

#### Health Coaching plus Activation Coaching (HC + AC).

—Participants in this arm received the above, plus Activation Coaching (AC). AC was developed by the UK brain tumor charity *brainstrust.* AC was targeted at coaching improvement in participants’ self-efficacy for managing fatigue. It was delivered by trained life coaches in two 1-hour sessions for each patient, with sessions separated by 4 weeks. Before each AC session participants completed the Patient Activation Measure (PAM)^28^ to inform the coach about the individual’s current level of knowledge, skills, and confidence about fatigue. AC then focused on promoting self-efficacy using Dilts’ Logical Levels^29^ in session one, and the FRAME^30^ and GROW^31^ models in sessions one and two (Supplementary Methods). During the trial this arm had been called simply “Patient Activation”. We have since re-named the intervention “Activation Coaching” to distinguish it more clearly from the original Patient Activation Measure to which it is unrelated.

### Primary Outcome

The primary outcome was feasibility of recruitment and retention. Recruitment feasibility required a recruitment rate “equivalent to 60 fatigued patients over 12 months”. This rate would permit a larger trial to recruit in a timely manner. Retention feasibility required all-cause attrition of under 40% at the study endpoint. There is no agreed critical threshold for attrition in QOL outcomes from cancer RCTs.^32^ In neuro-oncology attrition is often high: leading studies of QOL outcomes in highly impaired patients may report missing data frequency (even among surviving patients) of over 30% on outcome measures at four months.^33^ In such an understudied group we also anticipated a process of learning and systems development to iteratively minimize both avoidable attrition, and the impact of unavoidable attrition, which might inform future trials of interventions for brain tumor-related fatigue.

### Secondary and Exploratory Outcomes

Secondary outcomes were the acceptability of, and engagement with, the interventions among participants (evaluated by sessional attendance, diary inspection, and qualitative interviews). A further secondary outcome was to explore potential benefit via a quantitative analysis of outcome measures. In these exploratory outcomes participants completed the Brief Fatigue Inventory (BFI);^27^ the FACIT-Fatigue scale;^34^ the Hospital Anxiety and Depression Scale (HADS);^35^ the Psychological Outcome Profiles Questionnaire (PSYCHLOPS);^36^ the EQ-5D-5L;^37^ and the Addenbrooke’s Cognitive Examination III (ACE-III)^38^ at T0, T1, and T2. More detail on these measures, including comments on Minimal Important Clinical Differences for the two fatigue scales, is provided in Supplementary Methods.

### Qualitative Interviews

All those completing HC (+/− AC) were invited to participate in a semi-structured qualitative interview about their experiences and the perceived acceptability of the interventions. Interviews were guided by a template (Supplementary Methods), conducted by a qualitative researcher or allied health professional blinded to treatment allocation, audio-recorded, and transcribed. Interviews were audio-recorded, transcripts were analyzed using the framework method^39^: (1) Familiarization; (2) Construction of initial themes; (3) Indexing and sorting; (4) Review; and (5) Data summary.^39,40^ A realist approach^41^ provided a thematic framework (Context-Mechanism-Outcome) to analyze transcripts.

### Sample Size Calculation

Sample sizes were not calculated for this feasibility study.^42^ Results were used instead to calculate sample sizes for a future Phase II trial of efficacy (see Supplementary Methods).

### Randomization and Follow-Up

After obtaining informed consent a Research Assistant administered baseline measures. They accessed www.sealedenvelope.com to randomize with stratification by study site. It was not possible to blind participants or Research Assistants to allocation. Follow-up assessments were conducted 10 weeks (T1) and 16 weeks (T2) after randomization. The T1 time-point was chosen to measure symptoms shortly after the end of the 8-week intervention—assumed to represent the point of maximal signal, if any. The T2 time-point was chosen to allow a modest period of follow-up extending to a total of four months after baseline, to examine for early attenuation of any signal in this pilot study.

### Statistical Analyses: Primary Outcomes

For recruitment feasibility, the number of patients recruited was compared to the target rate of 60 patients per year. The number and proportion of patients retained to the T2 endpoint was compared to the target that at least 60% of patients should be retained.

### Statistical Analyses: Exploratory Outcomes

Box-and-whisker plots were produced for each outcome scale, broken down by treatment arm and separated by time-point. Changes to outcome scale scores from T0 (baseline) to T2 (endpoint) were compared among the study arms using a one-way ANOVA. Ninety-five percent confidence intervals were produced and two sided post-hoc comparisons made using Bonferroni (equal variances assumed) and Dunnett’s *C* (equal variances not assumed) statistics. A significance level of .05 was used. Waterfall plots were constructed to examine whether intervention arm effects clustered separately from control arm effects. Further statistical analysis detail is given in Supplementary Methods and below.

### Data Protection and Ethics

All participants gave written informed consent and GDPR-specific consent. The trial team used encrypted nhs.net email for all written communication. The trial was ethically approved by the South-East Scotland Research Ethics Committee 2 (18/SS/0025) and the local Caldicott Guardian.

## Results

### Primary Outcome: It was Feasible to Recruit and Retain Fatigued Brain Tumor Patients

**Figure 1.**
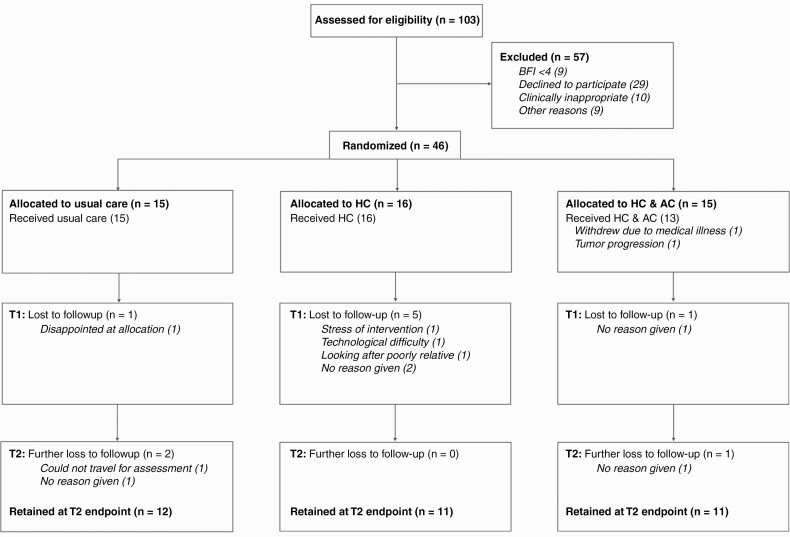
CONSORT Flow diagram. Patients who declined to participate (*n* = 29) were typically either unable to commit to regular coaching sessions (eg, due to traveling distance, family duties, or holidays), or unwilling to receive the interventions. The patients listed as “other reasons” (*n* = 9) gave no specific reason for not taking part.

**Figure 2.**
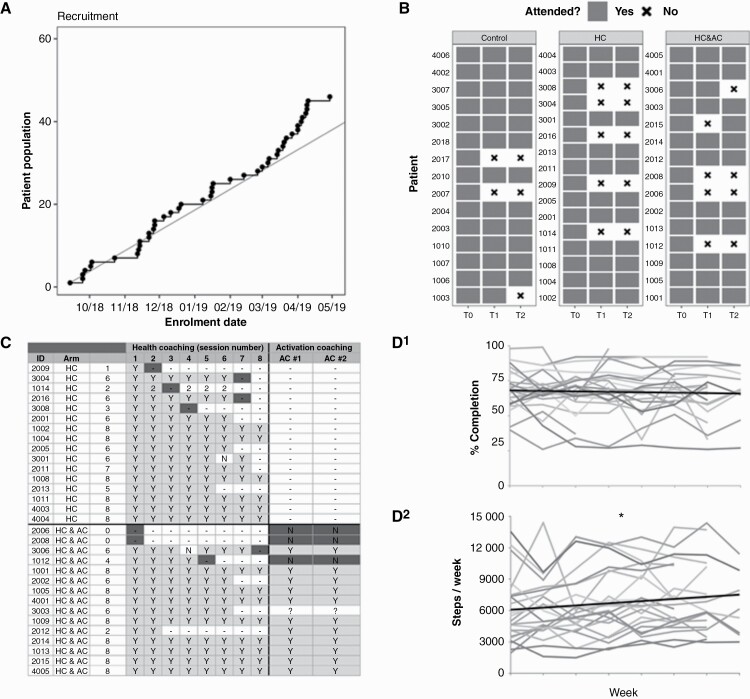
Lifestyle coaching was feasible to deliver and acceptable to fatigued patients. (A) Graph of cumulative recruitment between 16th Aug 2018 and 16th May 2019. The straight line is the target rate of 60 fatigued patients per year. (B) Individual patient attendance at T0, T1, and T2 outcome assessments. (C) Individual patient attendance at scheduled coaching sessions. Patient drop-out is marked in dark grey. (D^1^) % Diary completion (out of 106 possible fields per week). Each grey line is the course of one patient over 8 weeks of Health Coaching. The linear regression slope is shown by the thicker black line and demonstrates no reduction in diary completion over time. (D^2^) Steps per week measured by objective step counters. As shown by the thicker black regression line, step number increased slightly in the second half of HC compared to the first (paired-samples *t*-test *P* = .027). Individual level data for (D^1^) and (D^2^) are available from the corresponding author on request.

During a 9-month recruitment period (16th August 2018–15th May 2019) *n* = 103 primary brain tumor patients were screened from whom *n* = 46 fatigued patients were recruited (Figure 1, Table 1 and Supplementary Figure S4). This recruitment rate equated to 61 participants in 12 months, establishing recruitment feasibility (Figure 2A). At T2 endpoint 34/46 participants remained (retention = 74% [95% CI = 59, 86%], Figure 2B) establishing retention feasibility. Recruitment and retention were generally acceptable across sites and arms (Supplementary Table S1). The most frequent reason for drop-out was withdrawal of consent due for example to interventional burden, difficulty traveling, family commitments, and medical illness (for full details see Figure 1).

**Table 1.** Baseline demographic characteristics

	Control (*n* = 15)	Health coaching (*n* = 16)	Health + Activation coaching (*n* = 15)	Total (*n* = 46)	*P*
Age (mean, SD)	46 (10)	38 (10)	49 (13)	44 (12)	**.031** ^a^
Sex (M/F)	5/10	9/7	6/9	20/26	.414^b^
WHO tumor grade (*n*)					.951^c^
I	2	4	4	10	
II	6	5	4	15	
III	4	5	5	14	
IV	2	2	1	5	
Not known	1	0	1	2	
Tumor location*					.993^b^
Frontal lobe involvement	6	6	5	17	
Temporal lobe involvement	4	4	4	12	
Other location	6	8	8	22	
Tumor laterality					.728^b,d^
Left	5	5	7	17	
Right	8	9	7	24	
Both	1	1	1	3	
Midline	1	1	0	2	
Surgery**					.638^b^
Yes	15	14	14	43	
No	0	2	1	3	
Type of surgery (*n* = 43)					.815^b.d^
Biopsy	3	3	4	10	
Resection	12	11	9	32	
Data missing	0	0	1	1	
Chemotherapy**					.868^b^
Yes	8	10	9	27	
No	7	6	6	19	
Radiotherapy**					.313^b^
Yes	11	14	14	39	
No	4	2	1	7	
KPS					.599^b,d^
90–100	5	8	7	20	
70–80	9	8	6	23	
< 70	1	0	1	2	
Data missing	0	0	1	1	
Medications at baseline (*n*)*					NA^e^
EIAED	3	2	0	5	
Non-EIAED	8	8	9	25	
Antidepressant	8	8	5	21	
Non-opiate analgesia	3	2	1	6	
Opiate analgesia	4	1	2	7	
Vitamin supplementation	4	2	1	7	
Hormonal replacements	3	4	3	10	
Measures (mean, SD)					
BFI	7.0 (1.5)	6.4 (1.4)	7.1 (1.3)	6.8 (1.4)	.307^a^
FACIT-Fatigue	21.3 (8.5)	24.9 (10.0)	19.5 (7.5)	22.0 (8.8)	.233^a^
HADS-D	8.7 (3.6)	6.9 (3.7)	8.1 (3.4)	7.8 (3.6)	.369^a^
HADS-A	11.3 (5.2)	7.9 (4.5)	10.0 (4.6)	9.7 (4.8)	.143^a^
PSYCHLOPS	14.7 (3.1)	12.6 (3.6)	16.1 (3.3)	14.4 (3.6)	**.017** ^a^
EQ-5D health	12.3 (4.1)	10.1 (3.1)	10.9 (2.9)	11.1 (3.4)	.201^a^
EQ-5D VAS	53.1 (18.6)	55.4 (24.5)	50.2 (20.0)	53.0 (20.9)	.796^a^
ACE-III	86.7 (9.4)	89.8 (7.5)	89.1 (7.8)	88.6 (8.2)	.559^a^

All analytic tests on baseline demographic data were post-hoc. Significant *P* values are shown in bold.

*Totals may sum > 46 due to potential for one patient to fill multiple categories.

^**^For duration since surgery, chemotherapy, or radiotherapy in individual participants see Supplementary Figure S4.

^a^One-way ANOVA.

^b^Fisher’s Exact Test.

^c^Chi Square.

^d^Cells containing “1” or “0” excluded from statistical analysis.

^e^Data not amenable to Fisher’s or Chi Square tests.

In this small study the intervention arms were balanced on most characteristics, but participants randomized to HC were significantly younger. At baseline the groups also differed on the PSYCHLOPS outcome measure: participants in the HC arm reported better function and participants in the HC+AC arm reported poorer function on this scale, relative to controls (Table 1).

### Secondary Outcome: Lifestyle Coaching was Acceptable, Manageable, and Safe

#### Acceptability and engagement.

—Fifteen participants received Control and 31 patients were randomized to an intervention (Figure 1, HC *n* = 16, HC + AC *n* = 15). Two participants randomized to HC + AC dropped out for unrelated medical reasons before starting any intervention. Those remaining started HC on average 17 days (SD = 9, range = 2–36) after randomization, completing a median of seven sessions (IQR = 6–8, mode = 8, range = 1–8, Figure 2C). The first AC session occurred after a median of four HC sessions and the second after seven. All patients who received the first AC session also completed the second.

Participants returned a median of seven diaries (maximum = 8). Those remaining in the study throughout showed high rates of diary return (median = 7 diaries returned, mode = 8, range = 3–8), unlike those destined to drop-out (median = 0 diaries, mode = 0, range = 0–8) (Figure 2C). Each diary contained 106 individual cells to optionally complete. Among participants returning at least one diary (*n* = 25/31) mean cell completion was 67.2% (SD = 14.2%, range = 35–90%). Cell completion was similar between intervention groups and remained stable throughout the study (Figure 2D^1^) with no evidence of “diary fatigue” over time.

We examined step count data as an objective test of engagement with HC. In 23/31 participants returning step data, the mean weekly count was 6674 steps (SD = 2815, range = 2569–12 099), with no significant difference in frequency between intervention arms. In an exploratory post-hoc analysis there was a positive effect of coaching over time (mean steps in the first half of coaching = 6273/week [SD = 2834]; second half of coaching = 7031/week [SD = 2798], paired-samples *t*-test *P* = .027). Normalized to their performance in the first half of coaching, participants showed a 17% increase in physical activity in the second half (SD = 29%, range −21 to +83%, Figure 2D^2^). These modest improvements suggested that coaching could improve outcomes in fatigued patients.

Participants completed outcome questionnaires well. At T0 baseline (*n* = 46) questionnaire completion was 100% except for the FACIT-F (45/46 completed). At T1 (*n* = 36) questionnaire completion was 35/36 for all questionnaires, due to one participant missing the T1 appointment, except PSYCHLOPS (33/36) and ACE-III (34/36). At T2 (*n* = 34), questionnaire completion was 100% except for PSYCHLOPS (30/34), EQ-5D (33/34) and HADS (33/34). The completion of all potential questionnaires at all potential timepoints was 97.7% (907 actual versus 928 potential data points).

#### Qualitative feedback.

—Among patients who completed HC or HC + AC, *n* = 21 were interviewed in detail about their experience of the interventions. This sub-sample included 10 female and 11 male participants aged between 25 and 63 (mean age 44). Participants came from both the HC (*n* = 9) and HC + AC (*n* = 12) arms of the trial, and from all three sites: NHS GGC (*n* = 10), NHS Lothian (*n* = 8) and The Christie Hospital, Manchester (*n* = 3). They represented a spectrum of fatigue with moderate (*n* = 13) and severe (*n* = 8) levels of baseline BFI fatigue.

Overall acceptability was good although influenced by contextual factors, including patient expectations and attitudes to life, prior experience of making lifestyle changes, and the emotional and physical constraints of living with a brain tumor. The degree to which the coaching approach was perceived to “fit” with the individual’s beliefs and way of life was important to overall engagement with the interventions.

Some patients found the interventions compatible with their needs whereas others expressed needs that were unmet by the coaching approach. Potential mechanisms of change included pragmatic factors such as goal-setting and monitoring, and supportive factors such as the motivational “push” from coaches and family or friends. Many participants also reported improvements in general wellbeing and awareness of health behaviors. Some individuals reported specific improvements in fatigue including being “better able to cope” with fatigue. Full qualitative results will be reported elsewhere.

#### Adverse events.

—No adverse events were reported and no patients were withdrawn from the trial by study investigators.

### Exploratory Outcomes: Coaching Improved Fatigue and Mental Health

#### Fatigue.

**Figure 3.**
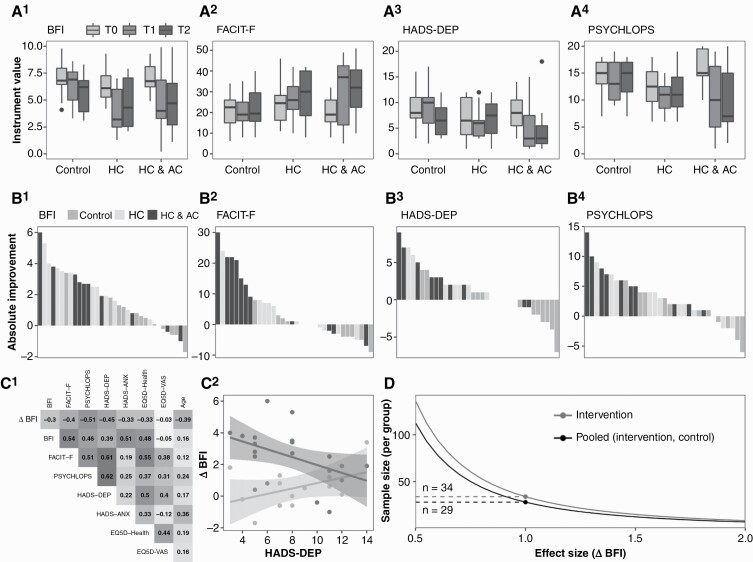
Lifestyle coaching showed a positive signal of effect on fatigue and QOL outcomes. (A1–4) Box-and-whisker plots for aggregate raw data for BFI, FACIT-F, HADS-D, and Psychlops. Lower raw scores represent improvement for all scales except the FACIT-F, in which higher scores represent improvement. The plots are grouped by trial arm, within which each time-point is represented a different shade of grey. (B1–4) Waterfall plots of individualized change in outcome scores (absolute difference) at T1, compared to T0, for BFI, FACIT-F, HADS-D, and Psychlops. The waterfall plots have been constructed such that a positive change represents clinical improvement at T1 in all plots including the FACIT-F. Note that the HC and HC + AC interventions tend to cluster in showing the strongest benefit. (C1) Correlogram comparing baseline T0 measures (*X*-axis along the top) with change in outcome scale scores at T1 (*Y* axis down the side), for the two coaching populations combined. Negative correlations suggest that as the T0 QOL measure worsens, clinical improvement at T1 lessens. In general, worsening baseline measures predicted less impact of coaching upon T1 BFI fatigue. (C2) Visualization of the negative correlation between worsening T0 depressive symptoms (left-to-right along the *X*-axis) and less improvement in BFI at T1, in patients receiving a coaching intervention (dark grey) versus controls (light grey). (D) Estimated sample size per group for a range of assumed differences in T1 BFI score between intervention and control. The plot shows two curves, one estimating the variance from Intervention alone and another with the pooled variance of Control and Intervention, the latter being smaller and yielding smaller sample sizes.

—At T1, both intervention arms showed statistically significant improvements in BFI scores relative to change in the control arm (T1–T0 BFI improvement over control: HC = 2.2 point improvement [95% CI 0.6, 3.8 points, Cohen’s *d* = 1.88], HC + AC = 1.8 [0.1, 3.4, *d* = 1.10], one-way ANOVA *P* = .004). FACIT-Fatigue scores also improved in both arms with clinical and statistical significance in the HC + AC arm (T1–T0 FACIT-Fatigue improvement over control: HC = 4.8 points [−3.7, 13.3, *d* = 0.47]; HC + AC = 12 [3.5, 20.5, *d* = 0.89]; one-way ANOVA *P* = .01) (Figure 3A^1,2^ and Table 2). In the HC + AC arm, FACIT-Fatigue improvement was sustained to endpoint (T2–T0 improvement on control: HC = 3.2 points [−5.4, 11.8, *d* = 0.49]; HC + AC = 9.9 [1.3, 18.5, *d* = 1.15]; one-way ANOVA *P* = .03). Two-way mixed ANOVA found significant interactions between arm and time for both fatigue scales (Supplementary Results). Waterfall plots of T1–T0 improvement suggested that both interventions had acute benefits on fatigue versus control with no consistent separation of the two active intervention groups (Figure 3B^1,2^ and Table 2).

**Table 2.** Change in pilot outcome measures at T1 and T2

Scale (range)	T0 baseline	T1 (vs T0)		T2 (vs T0)	
	Mean score (SD)	Mean change (SD)	One-way ANOVA *P*	Mean change (SD)	One-way ANOVA *P*
BFI (0–10)					
Control	6.6 (1.6)	−0.59 (1.3)	**.004**	−1.2 (1.5)	.374
Health Coaching	6.2 (1.4)	−2.71 (1.4)		−2.1 (2.6)	
Health + Activation Coaching	6.7 (1.1)	−2.26 (2.1)		−2.5 (2.1)	
FACIT-Fatigue (0–52)					
Control	21.5 (8.2)	−1.0 (4.5)	**.009**	+1.3 (5.1)	**.025**
Health Coaching	24.9 (10.3)	+3.8 (8.1)		+4.5 (9.1)	
Health + Activation Coaching	19.5 (7.5)	+11.0 (12.3)		+11.2 (9.7)	
HADS-D (0–21)					
Control	8.7 (3.6)	+0.85 (3.1)	**.009**	−1.2 (2.8)	.327
Health Coaching	6.9 (3.7)	−2.00 (2.4)		−0.8 (2.7)	
Health + Activation Coaching	8.1 (3.4)	−2.91 (3.1)		−2.6 (3.4)	
HADS-A (0–21)					
Control	11.3 (5.2)	−1.1 (2.1)	.235	−1.6 (3.4)	.333
Health Coaching	7.9 (4.5)	−0.0 (3.4)		+0.1 (4.5)	
Health + Activation Coaching	10.0 (4.6)	−2.1 (3.0)		−2.4 (3.6)	
PSYCHLOPS (0–20)					
Control	15 (3.1)	−0.67 (3.5)	**.007**	−1.1 (3.5)	**.018**
Health Coaching	13 (3.6)	−3.09 (3.5)		−2.4 (4.1)	
Health + Activation Coaching	16 (3.3)	−6.00 (4.0)		−6.1 (3.7)	
EQ-5D Health (5–25)					
Control	12 (4.1)	−0.54 (2.7)	.327	−2.18 (3.1)	.451
Health Coaching	10 (3.1)	−2.09 (2.3)		−1.82 (2.8)	
Health + Activation Coaching	11 (2.9)	−0.91 (2.6)		−0.73 (2.4)	
EQ-5D VAS (0–100)					
Control	53 (19)	+5.2 (25)	.202	+3.9 (20)	.374
Health Coaching	55 (25)	+6.5 (20)		+6.2 (20)	
Health + Activation Coaching	50 (20)	+21.2 (23)		+17.6 (31)	
ACE-III (0–100)					
Control	87 (9.4)	+3.00 (7.6)	.616	+3.1 (7.6)	.925
Health Coaching	90 (7.5)	+0.09 (8.5)		+1.8 (9.7)	
Health + Activation Coaching	89 (7.8)	+0.55 (6.6)		+2.7 (5.8)	

Significant *P* values are shown in bold.

#### Mental health.

—Depressive symptoms (HADS-D scores) improved at T1 in the HC + AC arm (Kruskal–Wallis *P* = .02, Dunnett’s post-hoc *P* = .01, Cohen’s *d* = 0.92) (Figure 3A^3, B3^ and Table 2). PSYCHLOPS scores improved in the combined HC + AC arm at T1 (Fig 3A^4, B4^ and Table 2, ANOVA *P* = .01, post-hoc Bonferroni *P* = .01, Cohen’s *d* = 1.5), sustained at T2 (ANOVA *P* = .02, Bonferroni *P* = .02, Cohen’s *d* = 1.7). No significant changes in HADS anxiety, EQ-5D Health Status, or ACE-III cognition were found (Table 2 and Supplementary Figure S5).

#### Severe depressive symptoms may limit potential coaching benefits.

—Correlation of baseline measures with BFI improvement at T1 were negative (Figure 3C^1^), suggesting that coaching had greater impact in participants who were relatively less impaired at baseline. As there were no clear differences of effect between the two intervention arms, we combined them to create one “grouped interventions” arm in exploratory modeling analyses. Linear modeling showed a mean T1 BFI improvement of 2.5 points (95% CI 1.8, 3.2) in the “grouped interventions” arm compared to the control arm (Supplementary Table S5). This model explained 28.6% of BFI score variation at T1 (adjusted *R*^2^ = 26.3%).

To explore whether depressive symptoms mediated this improvement, a second linear model considered baseline HADS-depression (HADS-D_T0_) scores in addition to allocation. In the “grouped interventions” arm, baseline HADS-depression was a significant explanatory variable for the change in BFI at T1 (Supplementary Table S5, Figure 3C^2^). As baseline HADS-depression increased there was less improvement on the BFI following an intervention. This improved model explained 42.5% (adjusted *R*^2^ = 36.8%) of the variance, suggesting that patients with lower depression scores at baseline may benefit more from lifestyle coaching for fatigue.

## Discussion

It is feasible to deliver lifestyle coaching interventions to fatigued brain tumor patients. Willingness to take part and remain in BT-LIFE was high and most participants engaged meaningfully with the coaching procedures. Qualitative analysis suggested that the compatibility of coaching with an individual’s prior experiences was a key mediator of engagement. There were favorable but preliminary signals of beneficial effects on fatigue, psychological outcomes, and depressive symptoms. Exploratory modeling suggested a limiting effect of higher depressive symptoms on the effect of coaching on fatigue. Our data suggest that patients who were moderately rather than extremely impaired, and who “believe” in a coaching approach, may be more likely to benefit—but much more study is needed.

### Limitations and Strengths

In this pilot RCT we did not record the number of patients who declined to be screened, nor did we measure ethnicity or tumor histological subtype. Follow-up duration was relatively short meaning that sustainability of the interventions is not known. Analyses were not powered or intended to test efficacy but to the extent that quantitative data can be weighed, outcome scales conflicted on whether improvements were seen in both intervention arms or sustained until the study endpoint; reasons for these conflicts are not clear and will require more data to unpick. Whether the interventions can be scaled beyond the study authors also remains open: it will benefit future studies to manualize each separate coaching session. The optimal timing for coaching, and mechanisms of action, if any, are not known and likewise questions for future studies. Therefore apart from the primary outcome of feasibility, much of the current output is preliminary.

In conducting the study, we became aware that “coaching” itself remained poorly defined. We established that it is feasible for fatigued patients to engage with people who call themselves coaches but did not examine which elements distinguish a coaching intervention from a more typical clinical intervention. What aspects of coaching are irreducible? What makes a good coach? Who is best placed to coach cancer patients in healthcare settings? Others may know the answers to these questions, but we do not. Exploring them may be helpful for future studies.

Alongside these limitations BT-LIFE had several novel or notable features. To our knowledge it is the first study to establish the feasibility of targeting highly fatigued brain tumor patients with complex interpersonal interventions. It is one of a handful of trials to report a signal of clinical and statistical effect on fatigue in these patients. The study blended private, charity, and health sectors to present a successful model of “multi-sectoral research”. Multisectoral research partnerships in which each sector has something valuable to the others have great potential for synergy, innovation, and new ways of delivering new interventions that may benefit patients.

### Results in Context

In neuro-oncology, there is mounting evidence from RCTs that psychostimulant medication is ineffective for fatigue and most secondary QOL outcomes,^11–16^ while two pilot RCTs of non-pharmacological interventions have reported preliminary improvements in fatigue and neurocognitive functioning.^23,26^ Although the effectiveness of any non-pharmacological strategy is yet to be proven for brain tumor patients, the wider oncology literature supports a shift in this direction: exercise, resistance training, core stability, psychological therapies, yoga, mindfulness, and hypnosis are listed among the therapies showing varying degrees of promise for fatigue in other cancer populations.^43^

Engagement with HC overall was good. Every patient who started AC continued to the second session. Most patients who were interviewed reported benefits on symptoms, lifestyle behaviors, or ability to manage ongoing fatigue. These insights suggest that fatigue is not an intractable or treatment-refractory problem: it may simply be that such a complex symptom is better suited to management by complex interventions.

### Next Steps

The next steps will study the essence, scalability, and efficacy of the coaching interventions piloted in BT-LIFE. A Phase II trial could formally manualize interventions, develop mechanisms to ensure blinding of outcome assessors, and perhaps stratify by symptom severity.

## Conclusion

BT-LIFE established that lifestyle coaching interventions are feasible to deliver to fatigued brain tumor patients. Coaching interventions were manageable, acceptable, and safe, with preliminary evidence of benefit on fatigue and mental health outcomes. Further trials are justified to test the efficacy of lifestyle coaching interventions in this highly impaired patient group.

## Supplementary Material

npac086_suppl_Supplementary_Data_S1

npac086_suppl_Supplementary_Data_S2

npac086_suppl_Supplementary_Figure_S1

npac086_suppl_Supplementary_Figure_S2

npac086_suppl_Supplementary_Figure_S3

npac086_suppl_Supplementary_Figure_S4

npac086_suppl_Supplementary_Figure_S5

npac086_suppl_Supplementary_Table_S1

npac086_suppl_Supplementary_Table_S5
