# Supplementary material for: Lifestyle coaching is feasible in fatigued brain tumor patients: A phase I/feasibility, multi-center, mixed-methods randomized controlled trial
Source: Neurooncol Pract. 2022 Oct 14;10(3):249–60. doi: 10.1093/nop/npac086 (PMC10180387; doi:10.1093/nop/npac086)
Supplement: npac086_suppl_Supplementary_Data_S1 [file npac086_suppl_supplementary_data_s1.docx]

**SUPPLEMENTARY METHODS**

CONTENTS

1. Health Coaching materials

1.1 Health Coaching protocol

1.2 Health Coaching initial consultation form

1.3 Lifestyle information recorded

1. Activation Coaching materials

2.1 Activation Coaching Protocol

2.2 Dilts’ Logical Levels model and sample questions (first session)

2.3 F.R.A.M.E. model and example approach (second session)

2.4 G.R.O.W. model and example approach (second session)

1. Outcome scales

3.1 Brief Fatigue Inventory

3.2 FACIT-Fatigue scale

3.3 Hospital Anxiety and Depression Scale

3.4 PsyCHLOPS

3.5 EQ-5D

3.6 Addenbrooke’s Cognitive Examination-III

1. Qualitative interview schedule
2. Statistical plan

5.1 Post-hoc analyses

1. Supplementary references

7. Final study protocol

**1. Health Coaching Materials (see also Fig S3 and S4)**

**1.1 Health Coaching Protocol**

Following informed consent and before the first Health Coaching appointment, participants were asked to start recording lifestyle information, using whichever method they choose, before their first Health Coaching appointment. A Health Coaching ‘Consultation Form’ (see below) was given to all participants to complete.

At the first Health Coaching appointment (45 minutes minutes – face to face in clinic)

- Discussed Consultation Form and explained DREEMS model.
- Discussed lifestyle data already gathered.
- Set goal(s) for week ahead.
- Participant and Health Coach arranged the next follow up appointment.

Between appointments

- Participants recorded lifestyle information using the standardised form. Information was recorded as often as possible but it was not mandatory to record it every day.

Follow-up appointments (30 minutes - optionally in clinic / home / phone / Skype)

- The participant’s Health Coach reviewed recorded lifestyle information recorded since the previous appointment, following the DREEMS model.
- New goal/s were set as appropriate. Participants were encouraged to maintain goal already in place from the previous appointments.
- Participants were able to ask questions and discuss difficulties.

**1.2 Health Coaching Consultation form**

“Before you have your first Health Coaching appointment, it would be helpful for me to know about your current health/lifestyle behaviours, as well as any goals you might have. Your Research Assistant, or carer/relative can help you complete this form if you would like. Once you have completed the form I will start to design a training plan to suit your unique needs. I look forward to working with you.

Your Health Behaviours and Lifestyle

Do you have any hobbies or interests?

How would you rate your current activity level? (Vigorous / Moderate / Mild)

How physically able do you consider yourself to be? (Very able / Less able / Not able)

Has having a brain tumour affected either your movement or balance? (Yes / No)

Your Medical History

Have you ever been diagnosed with any of the following?

- Heart Disease YES/NO
- High Blood Pressure YES/NO
- High Cholesterol YES/NO
- Diabetes YES/NO

Do you smoke tobacco products? YES/NO

Do you have any other health conditions or injuries that might impact your physical abilities? YES/NO (if yes please give details)

Are you currently taking any medication?

Health Goals

Can you think of any health goals you might want to work towards during your health coaching sessions? These goals are unique to you and your lifestyle. Examples might include, eating less processed food, drinking more water, being more active, etc.

Please list any health goals below.

Emergency contact

Please give details of your emergency contact.”

**1.3 Lifestyle Information recorded**

Participants recorded standardised lifestyle information into a daily home diary structured around Drink, Rest, Eating, Exercise, Movement and Stress (DREEMS).

**Lifestyle Information (DREEMS)**

**D**rink**:** The number of each daily fluid item (water, milk, juice, tea, coffee and alcohol).

**R**est: Total sleep time (hours per day).

**E**ating**:** Food items. Food items measured include Protein, Fruit, Vegetables, Dairy, Legumes, Nuts, Healthy Fats, Starches and Indulgent foods.

**E**xercise: Frequency and duration of exercise sessions.

**M**ovement**:** Total number of steps taken per day was recorded objectively by a wearable accelerometer. The data was written into the daily home diary by the participant.

**S**tress: Stress was self-reported using a simple three-stage scale; low/neutral/high.

**2. Activation Coaching Materials**

**2.1 Activation Coaching Protocol**

Following informed consent and allocation to this arm**,** the recruiting site Research Assistant discussed the participant’s preference of location of Activation Coaching - at home, Skype, telephone, or facetime. The Research Assistant liaised with *brainstrust* and the participant to arrange the time of the first session.

First Activation Coaching session

1. The Activation Coach met the participant as arranged, and discussed the aims and use of AC in the context of fatigue and the study.
2. The Coach administered the PAM-13 Patient Activation measure.
3. The Coach and the participant discussed the PAM-13 results to help the coach understand the degree of knowledge, skills and confidence the patient felt about managing fatigue.
4. The coach then led the participant through the semi-structured “Dilts’ Logical Levels” schedule with the goal of increasing the participant’s skills, knowledge and confidence to manage their own fatigue.
5. The Coach and participant jointly agreed suitable goal(s) for the participant to aim for.
6. The next coaching session date and location preference were set.

Between sessions

The Activation Coaches made notes of their session content on [nhs.net](http://nhs.net) and emailed them to the central study team for archiving. Participants receiving Activation Coaching were not asked to keep a diary of any specific actions between sessions.

Second Patient Activation session (c. 4 weeks after first)

1. There was a general discussion of progress since last session.
2. The Activation Coach administered the PAM-13 again, and discussed any changes between coaching sessions one and two.
3. The Coach then applied the FRAME (**F**eedback, **R**esponsibility, **A**dvice, **M**enu, and **E**fficacy) and GROW (**G**oals, **R**eality, **O**ptions, **W**ay-forward) approaches to enhance patient self-efficacy in managing their fatigue.
4. Any problems or difficulties were elicited to explore options and articulate ways forward.

**2.2 Dilts’'’ Logical Levels model and sample questions (first Activation Coaching session):**

Purpose

*What do you think Health Coaching aims to achieve?*

*Do you think it will have an effect on your fatigue?*

*What is your hope/desire to achieve by taking part in this project?*

Identity

*What do you see as your role in Health Coaching?*

*Do you consider yourself to be a person who is interested in their health?*

Values

*How important is it to you to complete Health Coaching to your best ability?*

*Do you believe that Health Coaching will help your fatigue?*

*Do you feel that your Health Coach is important in achieving your goals?*

Capabilities

*How confident are you in achieving the goals and tasks set out by your Health Coach?*

*What will you need to complete Health Coaching to your best ability?*

*What bits of Health Coaching are you looking forward to tackling the most/least?*

Behaviors

*Can you think of things you might do to help you complete Health Coaching?*

*Are there any current activities that might not be productive in completing Health Coaching?*

*Has the Health Coach suggested any activities that you’ve already thought about doing?*

Environment

*Who or what in your environment do you think will be important in achieving your goals?*

*Is there anything in your environment that you find particularly exhausting?*

*Can you think of things that help reinvigorate you?*

**2.3 F.R.A.M.E. model and example approach (second Activation Coaching session)**

**Feedback**: Discuss the lowest rated items on the PAM-13. *E.g.* If the participant registered a low score on Item 9, the coach might open a discussion of the perceived importance *to the participant* of knowing about different treatment options.

**Responsibility**: Assess the participant’s perceived personal responsibility in improving knowledge/confidence. *E.g.* If they do not perceive themselves to have responsibility, the coach might seek to increase their level by asking the participant about the pros and cons of their current approach.

**Advice**: Collaborative goal-setting.

**Menu**: Discuss the menu of options for self-management relating to issues discussed in *Feedback* section. Start with what the participant feels comfortable aiming for in the short term. Move on, if suitable, to longer-term goals.

**Efficacy**: Enhancement strategies for self-efficacy. Use previous successes to motivate new advances in their health. Break goals into attainable steps. How can they take the initial steps to achieve this? What support will they need? Who can help them?

**2.4 G.R.O.W. model and example approach (second Activation Coaching session)**

**Goal**: Discuss the goal from first session and any progress in achieving it.

**Reality**: What has happened in achieving the goal? Have they got any closer in certain aspects – has anything been put back? Is this goal still realistic? What has stepped in the way of the goal?

**Options**: What has changed/developed that we can apply to achieving the goal? Are there any recent developments? Brainstorm possible options to achieving goals. What is still a limiting factor in your recovery/ability to achieve your goals? What if this constraint was removed?

**Way forward**: What is still driving you to make these changes? What will you do now? When would you like to do it? What might set you back, and how would you tackle this?

**3. Outcome Scales**

**3.1 Brief Fatigue Inventory (BFI)**

The BFI is a nine-item fatigue scale measuring a single dimension that can be considered as the subjective report of fatigue severity. We selected it for use in this study because: a) it is validated for use in cancer patients [Mendoza 1999]; b) it has been used as a secondary outcome measure in an RCT of drug treatment for fatigue in patients with glioma [Lee 2016]; and c) pilot data from an earlier post-doctoral project using the BFI underpinned the successful grant application for the current trial.

Each item on the BFI is scored from 0-10 and the items averaged to generate an overall ‘fatigue severity score’ covering the previous 24 hours. Higher scores represent worse fatigue. To be eligible for BT-LIFE patients had to have a mean baseline fatigue severity of 4/10 or greater, indicating at least moderate fatigue. The BFI does not have a well-studied Minimal Important Clinical Difference (MICD), but the MICD for a different (non-averaged) 10-point fatigue scale has been estimated at approximately 2.4 points. [Schwartz 2002]

**3.2 Functional Assessment of Chronic Illness Therapy - Fatigue (FACIT-Fatigue)**

The FACIT-Fatigue is a 13-item fatigue sub-scale originally derived from the larger FACIT-F, which was validated in cancer patients with good internal consistency and test-retest reliability. [Yellen 1997] We used the FACIT-Fatigue in addition to the BFI because we were aware that it has been used as the primary outcome measure in RCTs of drug treatment for fatigue in patients with glioma. [Butler 2007, Lee 2016] We intended to gather experience using it and derive pilot data from the FACIT-F that would contribute to applications for further funding of larger trials.

After reverse scoring of certain items, FACIT-Fatigue score ranges from 0 to 52 with higher scores indicating less fatigue over the past seven days. Its reported Minimal Important Difference ranges between 3 and 8.3 points. [Nordin 2016]

**3.3 Hospital Anxiety and Depression Scale (HADS)**

The HADS is a 14-item screening questionnaire for anxiety and depressive symptoms, designed for use in medical populations. [Zigmond & Snaith 1983]. The full questionnaire comprises two seven-item subscales targeting anxiety and depression. The depressive sub-scale has been partially validated in glioma patients [Rooney 2013]. Each item is rated 0–3 with a maximum score for each sub-scale of 21. Higher scores indicate greater severity of anxiety or depressive symptoms over the preceding week.

**3.4 Psychological Outcome Profiles Scale (PSYCHLOPS)**

The PSYCHLOPS is a short four-item questionnaire developed to capture influences of QOL and wellbeing which are considered to have the most personal meaning and importance to individual patients. The questionnaire comprises ‘pre-treatment’ and ‘post-treatment’ scales. In the pre-treatment scale, the first question asks: ‘Choose the problem that troubles you most. Please write it in the box below’. Patients are then invited to score this problem on a six-point Likert scale (0-5) ranging from ‘not at all affected’ to ‘severely affected’. Further questions ask for a description and scoring of a second nominated problem, and of any functional impairment caused by the problems. Finally, the patient is asked to score their wellbeing. Following therapy the ‘post-treatment’ questionnaire reminds the patient of the problems they identified at the start, and invites them to score the same problems again. As well as an intuitive and individualized way to track the impact of treatment, the PSYCHLOPS displays particularly good sensitive to change. [Sales 2021] The total score is out of 20 with higher scores representing greater personalized distress.

**3.5 EQ-5D-5L and EQ-VAS**

The EQ-5D-5L is a five-item QOL screening measure surveying mobility, self-care, usual activities, pain, and anxiety/depression. [EuroQol] Each item has five levels of response ranging from “no problems” to “extreme problems”. Scores range from 5 to 20 where higher scores indicate poorer QOL. The accompanying EQ-VAS records the patient’s self-rated health that day on a Visual Analog Scale. The endpoints are labelled ‘The best health you can imagine’ and ‘The worst health you can imagine’. Scores on the VAS range from 0 to 100 where higher scores represent better QOL on the day of measurement.

**3.6 Addenbrooke’s Cognitive Examination-III (ACE-III)**

The ACE-III is a validated screening tool for cognitive deficits in frontotemporal dementia and Alzheimer’s Disease. [Hsieh 2013] It was chosen for use in BT-LIFE as a ‘compromise’ between briefer, more insensitive screening questionnaires (e.g. the MMSE [Meyers & Wefel 2003]) and the impracticality for our team of delivering full neuropsychological assessment. The ACE-III comprises five domains (Attention/Orientation, Memory, Verbal Fluency, Language, and Visuospatial) summing to a maximum possible score of 100. Higher scores represent better cognitive performance.

**4. Qualitative interview schedule**

**The interventions**

- Can you tell me about the intervention you received in this study and what you thought about it?
- What did you hope to achieve by taking part in the intervention?
  - Did you feel this happened?
    - Yes - What specific elements of the study contributed to this?
    - No - Why do you feel that you didn’t achieve these aspirations?
- Can you think of areas in the intervention that you liked the most?
- Were there any areas that you didn’t enjoy?
  - Which parts of the study, if any, do you think were difficult or came easily to you?
- In relation to your personal recovery, how well timed was the intervention?
  - Was your involvement proposed to you at the right time?
- How do you feel about the duration of the intervention?
  - Was the pace of intervention acceptable to your lifestyle?
    - No – which areas of your lifestyle were difficult to balance?
- How would you feel about completing a longer intervention period?
  - How much interaction with your health coach would aid this?
- How have you used the techniques you have learned in the interventions?
  - Have you used them in any other areas of your life?
    - Each element of DREEMS as probes (if not discussed)
- Was there anything that prevented your best participation in the study?
- Did anything or anyone particularly help you in completing the intervention?
  - Probe for carer influence

**Taking part in the study**

- What influenced you to take part?
  - Friends/family/doctor
  - Fatigue levels
- Before the study was introduced to you, did you feel your fatigue to be an issue?
  - Yes - how did you feel about combatting your fatigue?
    - Had you tried any other treatment strategies?
  - No – how do you feel about your fatigue now?
- Was there anything that worried you about taking part?
  - If yes – Could you think of anything that would have helped dispel these worries?
- Were there any times during the intervention that you thought of leaving?
  - What helped you keep going?
- How did you feel about travelling to the clinic/ having the team come to you?
- What did you expect from taking part in this study?

**Participant’s health**

- Can you tell me about how you are feeling about yourself at the moment, physically and mentally?
- How would you compare your health now to before the intervention?
  - Specific to fatigue/general health
  - Have your friends/family noticed this change?
    - How do you feel about those changes?

**Future outlook**

- How do you feel taking part in the study has influenced you, if at all?
  - In relation to fatigue/ non-fatigue symptoms
- Has the study changed the way you view your capacity to manage fatigue?
- How do you think being in the study might impact you in the next few months?
  - (prognosis dependent probes on further into future)
- How do you feel about continuing without the support of your health coach?
  - What do you think is going to be the easiest to maintain?
  - What is likely to prove difficult in this?
- Would you recommend the intervention to a fellow patient?
  - If yes – how could you guide them to aid their best participation?

**5. Statistical analyses.**

**5.1 Post-hoc analyses**

We conducted several exploratory post-hoc analyses. These analyses were identified by the study team at an away day focused on reviewing the trial and “learning lessons” for a better future study. By this point after two years of working on the trial, the team had learned more about the range of potential analyses permitted by the design. Following from this team discussion we analysed:

- Scale score changes from T0-T1, and T1-T2 were also explored using one-way ANOVA (using R function stats::aov()), to augment the more limited SAP analysis (T0-T2 only, which overlooked the important question of whether coaching had effects shortly after the end of the sessions). Post-hoc analyses were carried out using the Bonferroni or Dunnett’s methodology as appropriate (i.e., where data were identified as normal or not normal using stats::shapiro_test() and as implemented in the multicomp R packages.
- Fatigue outcomes were tested for an interaction between time and treatment arm, using two-way mixed ANOVA (using the R function stats::lm()) (leveraging all data from all three timepoints for a given scale).
- Waterfall plots were generated by calculating score changes for all outcome scales and visualised using the R package ggplot2 (visually examining whether the effect of interventions clustered together separately from that of control).
- Effect sizes (reported as Cohen’s D) for each scale and each trial arm were calculated using the function lsr::cohensD(). These effect sizes informed sample sizes estimations for a future trial, using equations for n_ANCOVA_ and n_CHANGE._ (Walters 2019).
- Spearman correlations were calculated between baseline variables and the impact of coaching interventions on BFI-measured fatigue at T1. These were visualised using a correlogram matrix (using the R function corrplot::corrplot()). From that analysis, strongly correlated baseline variables were then tested using ANCOVA for independent (beyond study arm allocation) prediction of BFI fatigue at T1; the ANCOVA models were build using the R function stats::lm(). The aim of this last analysis was to identify patient characteristics that may warrant stratification in a future trial.

6. **Supplementary References**

Butler JM Jr, Case LD, Atkins J, et al. A phase III, double-blind, placebo-controlled prospective randomized clinical trial of d-threo-methylphenidate HCl in brain tumor patients receiving radiation therapy. Int J Radiat Oncol Biol Phys. 2007; 69(5): 1496-501.

EUROQOL. <https://euroqol.org/eq-5d-instruments/> Accessed 3^rd^ October 2022.

Hsieh S, Schubert S, Hoon C, et al. Validation of the Addenbrooke's Cognitive Examination III in frontotemporal dementia and Alzheimer's disease. Dement Geriatr Cogn Disord. 2013; 36(3-4): 242-50.

Lee EQ, Muzikansky A, Drappatz J, et al. A randomized, placebo-controlled pilot trial of armodafinil for fatigue in patients with gliomas undergoing radiotherapy. Neuro Oncol. 2016 Jun; 18(6): 849-54.

Mendoza TR, Wang XS, Cleeland CS, et al. The rapid assessment of fatigue severity in cancer patients: use of the Brief Fatigue Inventory. Cancer. 1999 Mar 1; 85(5): 1186-96.

Meyers CA, Wefel JS. The use of the mini-mental state examination to assess cognitive functioning in cancer trials: no ifs, ands, buts, or sensitivity. J Clin Oncol. 2003 Oct 1;21(19):3557-8.

Nordin Å, Taft C, Lundgren-Nilsson Å, et al. Minimal important differences for fatigue patient reported outcome measures-a systematic review. BMC Med Res Methodol. 2016 May 26; 16: 62.

Rooney AG, McNamara S, Mackinnon M, et al. Screening for major depressive disorder in adults with cerebral glioma: an initial validation of 3 self-report instruments. Neuro Oncol. 2013 Jan; 15(1): 122-9.

Sales C, Faísca L, Ashworth M, et al. The psychometric properties of PSYCHLOPS, an individualized patient-reported outcome measure of personal distress. J Clin Psychol. 2021 Nov 20. doi: 10.1002/jclp.23278.

Schwartz AL, Meek PM, Nail LM, et al. Measurement of fatigue. determining minimally important clinical differences. J Clin Epidemiol. 2002 Mar; 55(3): 239-44.

Walters SJ, Jacques RM, Henriques-Cadby IBA, et al. Sample size estimation for randomized controlled trials with repeated assessment of patient-reported outcomes: what correlation between baseline and follow-up outcomes should we assume? Trials. 2019; 20(1): 566.

Yellen SB, Cella DF, Webster K, et al. Measuring fatigue and other anemia-related symptoms with the Functional Assessment of Cancer Therapy (FACT) measurement system. J Pain Symptom Manage. 1997 Feb; 13(2): 63-74.

Zigmond AS, Snaith RP. The hospital anxiety and depression scale. Acta Psychiatr Scand. 1983 Jun;67(6):361-70.

**7 Final study protocol
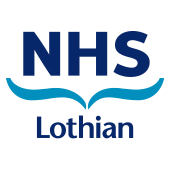
**




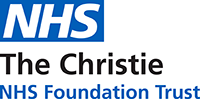

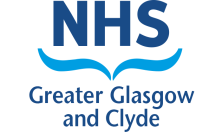

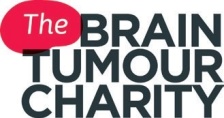


**BT-LIFE**

**B**rain **T**umours, **L**ifestyle **I**nterventions, and **F**atigue **E**valuation: a multi-centre, feasibility, Randomised Controlled Trial

Protocol v3.0 ( 4^th^ June 2019)

| **ISRCTN:** | ISRCTN17883425 |
| --- | --- |
| **REC reference:** | 18/SS/0025 |

**Main Sponsor**: NHS Lothian

**Co-sponsor:** The University of Edinburgh

**Funder:** The Brain Tumour Charity

**Trial Coordinating Centre:** Scottish Clinical Trials Research Unit, Edinburgh.

**Protocol Authors:** Dr. Alasdair Rooney (Chief Investigator, NHS Lothian)

Mrs. Tracy McEleney (Scottish Clinical Trials Research Unit)

Mrs. Michelle Welsh (Scottish Clinical Trials Research Unit)

Protocol authorised by:

Name: Dr Alasdair Rooney Role in Trial: Chief Investigator

Signature: Date: 4^th^ June 2019

**KEY CONTACTS**

**Chief Investigator:**

Dr. Alasdair Rooney

NHS Lothian / University of Edinburgh

Royal Edinburgh Hospital

Morningside

Edinburgh EH10 5HF

[ally.rooney@ed.ac.uk](mailto:ally.rooney@ed.ac.uk)

**Sponsor:**
NHS Lothian

c/o Kenny Scott

The Queen’s Medical Research Institute

47 Little France Drive

Edinburgh EH16 4TJ

[kenneth.scott@nhslothian.scot.nhs.uk](mailto:kenneth.scott@nhslothian.scot.nhs.uk)

**Co-Sponsor:**

The University of Edinburgh

c/o Jo-Anne Robertson

The Queen’s Medical Research Institute

47 Little France Drive

Edinburgh EH16 4TJ

[resgov@accord.scot](mailto:resgov@accord.scot)

**Funder:**

The Brain Tumour Charity

Registered Office

Hartshead House

61-65 Victoria Road

Farnborough

Hampshire GU14 7PA

**Trial Coordination:**

Scottish Clinical Trials Research Unit (SCTRU)

NHS National Services Scotland

Gyle Square, 1 South Gyle Crescent, Edinburgh EH12 9EB

[NSS.BT-Life@nhs.net](mailto:NSS.BT-Life@nhs.net)

**Trial Statistician:**

Richard Dobbie

NHS National Services Scotland

Gyle Square, 1 South Gyle Crescent, Edinburgh EH12 9EB

[NSS.BT-Life@nhs.net](mailto:NSS.BT-Life@nhs.net)

**Edinburgh site:**

Dr. Robin Grant (Principal Investigator, Edinburgh)

Edinburgh Centre for Neuro-Oncology

Western General Hospital

Crewe Road (south)

Edinburgh EH4 2XU

[robin.grant@nhslothian.scot.nhs.uk](mailto:robin.grant@nhslothian.scot.nhs.uk)

**Glasgow sites:**

Prof. Anthony Chalmers (Principal Investigator, Glasgow)

[anthony.chalmers@gla.ac.uk](mailto:anthony.chalmers@gla.ac.uk)

Beatson West of Scotland Cancer Centre

Gartnavel General Hospital

1053 Great Western Road

Glasgow G12 0YN

and

Queen Elizabeth University Hospital

1345 Govan Road

Glasgow

G51 4TF

**Manchester site:**

Dr. Catherine McBain (Principal Investigator, Manchester)

The Christie NHS Foundation Trust

Wilmslow Road

Withington

Manchester M20 4BX

[catherine.mcbain@christie.nhs.uk](mailto:catherine.mcbain@christie.nhs.uk)

**Qualitative research sub-study:**

Prof. Mary Wells (Principal Investigator, qualitative sub-study)

Nursing, Midwifery and Allied Health Professionals Research Unit

University of Stirling

Stirling FK9 4LA

[mary.wells@stir.ac.uk](mailto:mary.wells@stir.ac.uk)

**1. INTRODUCTION 7**

**1.1 Background & Trial Rationale 7**

**1.2 Strategic importance of this research 8**

**2. TRIAL OBJECTIVES 8**

**2.1 Primary Outcome 8**

**2.2 Secondary Outcome 8**

**2.3 Objectives and Key Deliverables 8**

**3. TRIAL DESIGN 9**

**3.1 General Design 9**

**3.2 Inclusion Criteria 9**

**3.3 Exclusion Criteria 9**

**3.4 Recruitment/Setting 9**

**3.5 Randomisation Codes 10**

**3.6 Withdrawal of Subjects 10**

**4. TREATMENT INTERVENTION 10**

**4.1 Treatment Schedule 11**

**4.2 Sampling time-points 12**

**4.3 Study materials 13**

**4.4 Concomitant Therapy 13**

**5. SAFETY MONITORING 13**

**5.1 Definitions 14**

**5.2 Expected events 14**

**5.3 Recording of Adverse Events 14**

**5.4 Recording and Reporting of Serious Adverse Events 15**

**5.5 Pregnancies 15**

**6. DATA MANAGEMENT 15**

**6.1 Data Collection 15**

**6.2 Record Keeping and Archiving 16**

**7. STATISTICS 16**

**7.1 Sample Size 16**

**7.2 Power considerations 16**

**7.3 Exit strategy to a definitive trial 17**

**7.4 Analysis Plan 17**

**7.5 End of Study 17**

**8. ACCESS TO SOURCE DATA/ DOCUMENTS 17**

**9. QUALITY CONTROL AND QUALITY ASSURANCE 17**

**9.1 Monitoring Visits 17**

**9.2 Data Monitoring Committee/ Trial Steering Committee 18**

**10. ETHICAL CONSIDERATIONS 18**

**10.1 Participant Confidentiality 18**

**10.2 Informed Consent 18**

**11. RESEARCH GOVERNANCE 19**

**12. FINANCING AND INSURANCE 19**

**13. PUBLICATION POLICY 19**

**14. REFERENCE LIST 20**

**Appendix 1a – Investigator Statement (SCTRU Copy) 23**

**Appendix 1b – Investigator Statement (Investigator Copy) 23**

**Appendix 1c - The Principles of ICH Good Clinical Practice 25**

**Appendix 1d - Schedule for Health Coaching intervention 26**

**Appendix 1e - Schedule for Patient Activation intervention 28**

**GLOSSARY OF ABBREVIATIONS**

| AE | Adverse Event |
| --- | --- |
| CaCTUS | Cancer Clinical Trials Unit, Scotland |
| CI | Chief Investigator |
| CRF  DSUR | Case Report Form  Development Safety Update Report |
| GCP | Good Clinical Practice |
| IB | Investigator Brochure |
| ICH | International Conference on Harmonisation |
| IDMC | Independent Data Monitoring Committee |
| IMP | Investigational Medicinal Product |
| MHRA | Medicines and Healthcare products Regulatory Agency |
| MREC | Main Research Ethics Committee |
| SAE  SCTRU | Serious Adverse Event  Scottish Clinical Trials Research Unit |
| SDV | Source Data Verification |
| TMG | Trial Management Group |
| TSC | Trial Steering Committee |

**TRIAL SUMMARY**

| Protocol ID | BT-LIFE |
| --- | --- |
| Protocol Title | Brain Tumours, Lifestyle Interventions and Fatigue Evaluation: a multi-centre, feasibility, Randomised Controlled Trial. |
| Development Phase | Feasibility Randomised Controlled Trial |
| Study Aims | In fatigued adult outpatients with a primary brain tumour, and in the setting of a multi-centre randomised controlled trial:   1. Assess the feasibility of delivering a structured lifestyle intervention (Health Coaching) and behavioural intervention (Patient Activation); 2. Determine their acceptability to patients and manageability for professionals; 3. Develop systems and pilot outcome measures for definitive RCTs of these interventions. |
| Primary Outcome | The feasibility of delivering Health Coaching and Patient Activation to fatigued patients with a primary brain tumour. Feasibility will be assessed by meeting a priori defined standards for recruitment and retention as follows:   - Recruitment will be feasible if we can recruit at a rate equivalent of 60 fatigued brain tumour patients per 12 months - retention will be feasible if total attrition at T2 (endpoint) is less than or equal to 40%. |
| Secondary Outcomes | 1. The acceptability of the interventions to patients. 2. The manageability of the interventions for professionals. 3. The development of systems and piloting outcome measures for future definitive RCTs of the interventions for fatigued brain tumour patients, including determination of mean change in outcome scale scores in each arm. |
| Study Design | Multi-centre, feasibility Randomised Controlled Trial with 16 weeks follow-up. |
| Patient Accrual | Eligible patients will be randomised to one of three study arms: control (n=20); Health Coaching (n=20); Health Coaching plus Patient Activation intervention (n=20) |
| Analysis | Will be performed when all patients have completed their assessments at the end of the 16 week follow up and all data has been cleaned and database finalised. |

# 1. INTRODUCTION

## 1.1 Background & Trial Rationale

Each year, more than 10,000 adults in the UK are diagnosed with a primary brain tumour.^1^ Many or most of them experience fatigue, as evidenced by consistent reports of fatigue prevalence of between 40-70%.^2,3^ In turn up to forty percent^4^ of these fatigued brain tumour patients regard it as ‘severe’, which strongly and independently reduces multiple domains of quality of life.^5,6^ Fatigue is in other words, a problem both of high frequency and of high impact for people living with a brain tumour. The causes of fatigue are however multi-factorial and vary between individuals.^7^ As a result complex interventions such as structured lifestyle or behavioural interventions have been proposed as treatment for fatigue.^8^ These non drug-based treatments focus on changing aspects of the patient’s lifestyle or behaviour. They have shown some efficacy in treating fatigue in patients with cancer arising out-with the central nervous system (CNS).^9,10^

It is therefore plausible that structured lifestyle and behavioural interventions could be effective treatments for fatigue in patients with a brain tumour. However two problems with this hypothesis must be addressed. The first is that the conclusions of prior studies may not apply to patients with a brain tumour because these studies have largely recruited patients with cancer arising out-with the CNS. Brain tumours by contrast inhabit a privileged location and may directly alter cognition, physical function, personality, and/or seizure threshold. These relatively ‘brain-tumour specific’ co-morbidities could subvert strategies known to be effective in other cancer patient groups. The natural solution to this problem is to study how to treat fatigue in patients with brain tumours specifically.

The second problem is that most prior studies included patients with little or no fatigue at baseline. They cannot therefore directly answer the clinically relevant question, which is how best to treat individuals with clinically significant fatigue.^8^ Here the natural solution is to recruit and study patients with clinically significant fatigue. The few studies to do so suggest that complex interventions can be delivered effectively in fatigued patients.^11,12^ But specifically which structured lifestyle and behavioural interventions could be studied?

A structured lifestyle intervention: Health Coaching

Health Coaching is a lifestyle intervention which targets basic elements of diet, exercise, sleep and stress. It has been developed and delivered in the community for several years by our collaborator.^13^ Participants monitor their dietary intake, movement, rest and stress levels daily and in a structured way. Working in partnership with the Health Coach - an appropriately trained and qualified practitioner such as a personal trainer or physiotherapist - patients are supported to make incremental positive changes to their lifestyle. Because similar lifestyle interventions have been shown to be effective in treating fatigue in other cancer populations^10,14^, we hypothesise that Health Coaching may be a effective treatment for fatigue in brain tumour patients.

A structured behavioural intervention: Patient Activation

Patient Activation (PA)^15^ is a behavioural intervention which leverages the individual’s knowledge and confidence so that they are ‘activated’ to self-manage their condition. Importantly, PA captures not only the patient’s *beliefs* about their ability to self-manage but also the *likelihood* that they will put these beliefs into action. Empowering patients through the framework of PA is thought to improve autonomy, quality of life, patient satisfaction, and cost-effectiveness.^16^ We hypothesise that Patient Activation could empower fatigued brain tumour patients to make and maintain lifestyle changes suggested by a Health Coach, potentially improving fatigue more than Health Coaching alone.

We wished to determine the level of existing evidence for these or similar lifestyle and behavioural interventions. To do this we systematically reviewed the evidence for effective interventions for fatigue in patients with a brain tumour.^17^ We found that nearly all eligible RCTs examined psychostimulant drug treatments, such as methylphenidate or modafinil ^(e.g.)18-20^ We found limited evidence that cognitive rehabilitation may improve mental fatigue as a secondary consequence of treating cognitive impairment.^21,22^ However, we found no high-quality studies of *any* non-pharmacological strategies such as lifestyle or behavioural interventions. Nearly all trials were further compromised by extending eligibility to non-fatigued patients. We concluded that there was a clear need for studies that evaluate structured lifestyle and behavioural interventions for significant fatigue in people living with a brain tumour.

In this respect candidate studies would ideally meet some important criteria.

- They would ensure *clinical significance* by recruiting patients with high levels of fatigue at baseline.
- Given the limited state of current knowledge they would focus first and appropriately on studying the *feasibility* of delivering interventions in a Randomised Controlled Trial.
- They would show *novelty* by studying interventions previously unstudied in this population.
- They would *add value* by including a qualitative sub-study to understand the acceptability of interventions, alongside their feasibility.
- *Credibility* would be assured by the involvement of an accredited Clinical Trials Unit.
- The *exit strategy* would be clear: the feasibility study would develop systems in sufficient centres to recruit plausibly to a definitive trial.
- Such studies would ideally be run by a *collaborative and expert team* with a track record in neuro-oncology symptoms research.

## 1.2 Strategic importance of this research

The strategic vision of this study is to move towards better treatment for brain tumour-related fatigue. Nearly two-thirds of brain tumour patients experience fatigue, with 40% reporting that it has affected them severely.^4^ Fatigue is regarded as a ‘top ten’ clinical research priority in people with a brain or spinal cord tumour, by the UK James Lind Alliance (JLA) Priority Setting Partnership in Neuro-oncology.^23^ More broadly by focusing on aspects of holistic assessment, lifestyle, late effects, and survivorship care, this study also reflects current thinking in the cancer field. Our approach aligns with the new Cancer Strategy in England, the Transforming Cancer After Treatment programme in Scotland, and the National Cancer Survivorship Initiative. This proposal directly targets a top priority of patients, charities, policymakers, and the wider research community.

# 2. TRIAL OBJECTIVES

## 2.1 Primary Outcome

The feasibility of delivering Health Coaching and Patient Activation to fatigued patients with a primary brain tumour.

Feasibility will be assessed by meeting a priori defined standards for recruitment and retention as follows:

- Recruitment will be feasible if we can recruit at a rate equivalent of 60 fatigued brain tumour patients per 12 months
- Retention will be feasible if total attrition at T2 (endpoint) is less than or equal to 40%.

## 2.2 Secondary Outcome

1. The acceptability of the interventions to patients.
2. The manageability of the interventions for professionals.
3. The development of systems and piloting outcome measures for future definitive RCTs of the interventions for fatigued brain tumour patients, including determination of mean change in outcome scale scores in each arm.

## 2.3 Objectives and Key Deliverables

We will obtain ethical and centre-specific approval for the study; gain access to neuro-oncology adult outpatient clinics; recruit 60 fatigued brain tumour patients from four centres; appraise the feasibility of Health Coaching and Patient Activation in fatigued participants; pilot outcome measures of fatigue impairment, functional impact, and efficacy of self-management; pilot measures of the confounding variables of mood change and cognitive impairment; pilot health economic measures; and gain a deeper understanding of which components of the interventions are acceptable to whom and why,. By doing so we will increase patient enrolment

into early-phase clinical trials, while laying the ground for a definitive trial of non-pharmacological interventions for fatigue in people living with a brain tumour.

# 3. TRIAL DESIGN

## 3.1 General Design

BT-LIFE is a multi-centre feasibility Randomised Controlled Trial, summarised schematically here (Figure 1). It is designed in line with guidance on developing complex interventions from the MRC^24^.

##
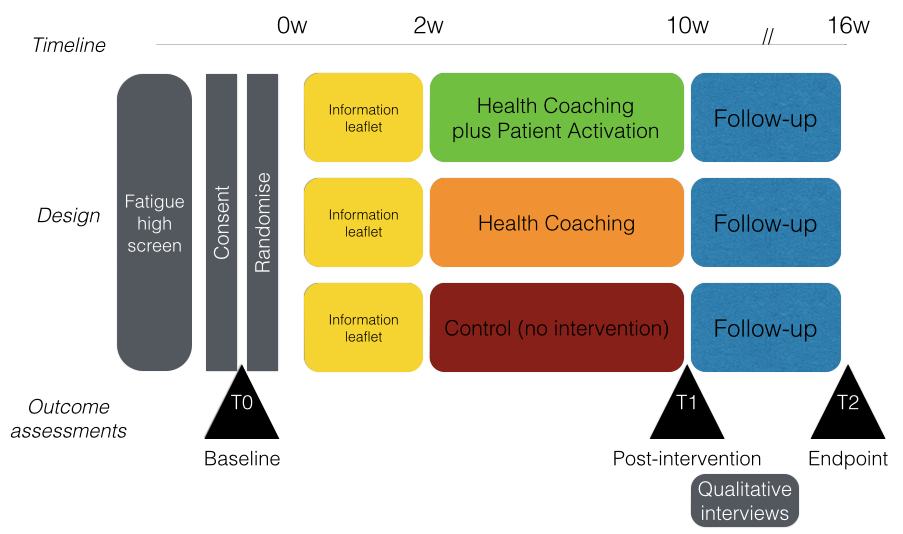


***Figure 1:*** *Schematic overview of the Brain Tumours, Lifestyle Intervention and Fatigue Evaluation (BT-LIFE) multi-centre feasibility Randomised Controlled Trial.*

## 3.2 Inclusion Criteria

1. Patients aged 18 and above;
2. Diagnosed with any primary brain tumour;
3. >3 months post-completion of chemotherapy/radiotherapy;
4. Clinically and radiologically stable, as defined by no evidence of disease progression at most recent clinic appointment;
5. Moderate or severe fatigue (Brief Fatigue Inventory score ≥4/10, indicating at least ‘moderate’ severity of fatigue over the previous week) - criterion for Randomisation.

## 3.3 Exclusion Criteria

1. Radiological or clinical concern at most recent appointment over disease progression;
2. Significant cognitive impairment, dysphasia, or visual impairment limiting ability to complete questionnaires;
3. Patients unable to give informed consent, or who are unable or unwilling to comply with interventions.

## 3.4 Recruitment/Setting

Patients will be recruited from neuro-oncology outpatient clinics in Edinburgh, Glasgow, and Manchester. Patients due to attend clinic and who meet inclusion criteria 1-3 above will be identified ahead of time. The Patient Information Sheet (PIS) will either be posted to them together with a covering letter from their usual treating clinician or administered during their clinic appointment. Patients will be given sufficient time to consider information about the study. Patients who have completed the questionnaire (and who have moderate-severe fatigue) will be asked by a member of their usual clinical team if they are interested in taking part in the study

Patients who score ≥4/10 on the BFI will be eligible to continue in the full study. In all sites they will then be introduced to a Research Assistant or Research Nurse who will obtain informed consent from the patient

The Baseline Assessment (T0) will then be conducted. Follow-up Assessments (T1 and T2) will be arranged according to preference: either via further attendance at outpatient clinics, or by posting forms to participants to complete at home and return in a stamped addressed envelope.

Immediately after the Baseline Assessment, participants will be Randomised.

## 3.5 Randomisation Codes

After ensuring that the patient meets all the eligibility criteria and has consented to participate, the RA/RN will log onto [www.sealedenvelope.com](http://www.sealedenvelope.com) to randomise. An identification number will be issued which should be used in all correspondence.. Participants will be randomised to one of three study arms:

- Control (n=20);
- Health Coaching (n=20);
- Health Coaching plus Patient Activation intervention (n=20).

These group sizes reflect national guidelines on appropriate group sizes for a feasibility study.^25^

Following randomisation, the participant’s GP will be informed that they are taking part in the study.

It may be possible for participants to be recruited into other clinical trials, but this should be discussed with the CI, via SCTRU, before this is considered.

## 3.6 Withdrawal of Subjects

In recognition that fatigued participants may find Health Coaching tiring without wishing to withdraw from the study entirely, we will use a two-level framework for withdrawing consent.

Participants may partially withdraw consent at any point without having to give a reason (by withdrawing from active treatment in the study, for instance any participant who may find Health Coaching too onerous) and may continue to be followed up per protocol if they wish.

Participants may also fully withdraw consent at any point without having to give a reason. Data that has been gathered up to that point will be censored at the point of withdrawal, unless the participant wishes further that none of their data gathered at any time should be used, in which case it will be removed from study records.

Any participant who indicates to us that they wish to withdraw consent will be treated according to the “withdrawal of consent” flowchart (see supporting documents).

# 4. TREATMENT INTERVENTION

(Post-Randomisation):

**Arm 1:**

Control (n=20). Participants randomised to the control arm will continue to receive the highest possible standard of care and support from their neuro-oncology team, who will be informed that the patient has high fatigue. The participant will also receive high-quality written information on how to manage fatigue (see Fig 1 and supporting documents). They will then be followed up at T1 and T2.

**Arm 2:**

Health Coaching (n=20). Participants randomised to this arm will be treated as per Arm 1, but in addition will receive Health Coaching. They will be given an initial consultation form to complete. They will then receive a standardised first appointment with a trained Health Coach, in a clinical setting, by arrangement to suit. This initial face-to-face appointment will cover 1) consultation form review; 2) Measures (blood pressure, resting heart rate, height, weight); 3) Muscle Activation exercises 4) Review of guidance videos; 5) Goal setting until the next appointment. Participants will be given a fitbit-style monitor to wear for the duration of the study.

Participants will then record standardised information about their lifestyle, on paper using a custom-designed diary form. The following information (the ‘DREEMS’ approach) will be gathered as often as possible (max daily) with assistance from the patient’s carer/relative if desired:

**D**rink. Caffeine, alcohol, water (number of each kind of drink per day, self-report)

**R**est. Total sleep (hours per day, self-report)

**E**ating. Various food groups (portions per day, self-report)

**E**xercise Aiming 3x30 minute sessions per week (self-report)

**M**ovement. Total number of steps taken per day (objective recording byfitbit-style monitor ).

**S**tress. Simple three-stage scale; low/neutral/high (self-report)

Health Coaching will be delivered for eight weeks. Over this period participants will receive up to 8 sessions lasting up to 45 minutes each. Sessions will be delivered by telephone, Skype, Facetime, or in clinic, according to individual preference. At each treatment session the participant and Health Coach will review and set goals to incrementally change lifestyle areas according to need. This kind of semi-structured but flexible approach is consistent with complex interventions shown previously to be efficacious for treating fatigue in cancer, such as exercise^9^, cognitive behaviour therapy^11^, or yoga.^12^ We will take steps to maximise parity of the intervention at all centres.

**Arm 3:**

Health Coaching plus Patient Activation intervention (n=20). Participants randomised to this arm will be treated as per Arm 2, but will also receive a Patient Activation (PA) intervention. In this intervention a trained PA coach will meet the participant and conduct a standardised semi-structured interview. These trained coaches will be supplied by *brainstrust*, a UK brain tumour charity with considerable experience in the field of personal coaching, and existing in-reach to all four study centres. *brainstrust* will ensure that all coaches delivering this intervention are trained to deliver it in a standardised way.

At the PA interviews participants will complete the Patient Activation Measure (PAM). The PAM is a 13-item measure^26^, validated in patients with cancer,^27^ that assesses Patient Activation. The focus of this short tool is to provide an initial measure of the participant’s knowledge, skill and confidence for self-managing their fatigue. The coaching intervention will then incorporate and leverage this information using the theoretical underpinning of Dilts model of Logical Levels^28^, which helps one understand one’s health status and make choices about what to do. They will coach the participant to appropriately leverage their activation to achieve their desired outcomes more effectively. Participants will be offered a second PA interview, identical in structure to the first, after a further four weeks.

## 4.1 Treatment Schedule

Please refer to Figure 1. In addition:

**Acceptability:** We will conduct qualitative interviews in a sub-study of 24 participants who were randomised to Health Coaching with or without Patient Activation. These interviews will be co-ordinated by a researcher appointed at the University of Stirling. They will be held within three weeks of the T1 assessment, and will be conducted in all four centres. To ensure reliability of interpretation and coding, data collection and analysis will be supervised by co-applicant MW, a senior and experienced qualitative researcher.

We will use a purposive maximum variation sampling strategy to ensure sample diversity. In this sampling strategy we will consider factors that may be important to the experience of fatigue, for instance sex, age, tumour type, severity of symptoms, whether the intervention included PA or not, type of health coach, and geographical location. We will invite participants to be interviewed in the company of their primary carer and in a location of their preference (e.g. at home). Interviews will investigate participants’ experience of Health Coaching. Specifically we will study barriers and facilitators to achieving goals, whether participants found the intervention(s) acceptable or not, and why, and how their experience of fatigue was affected.

These qualitative interviews will draw upon the concept of self-efficacy as a theoretical framework.^29^ This theory proposes that perceived ability to manage symptoms is important to achieving optimal symptom management. Self-efficacy is a key concept in the self-management of symptoms in cancer and other chronic illnesses.^30^ All interviews will be digitally-recorded on an NHS-encrypted device, transcribed and analysed according to the constant-comparative technique embedded within the overall “Framework” method.^31^ Analysis will be facilitated by the use of text management software.

**Manageability:** The Health Coaches and Patient Activation coaches will record referral waiting times, number and duration of assessments, number and duration of follow-ups, where participants prefer them to occur, number of contacts and their nature, time spent travelling on-study, and total hours spent on-study.

**Developing systems and piloting outcome measures:** In line with MRC guidance^24^ we will take the opportunity to develop and pilot systems and potential outcome measures for a future definitive trial. We will develop and use a structured clinical interview for fatigue, alongside the Brief Fatigue Inventory^32^ and the Functional Assessment of Chronic Illness Therapy-Fatigue (FACIT-F)^33^, to study the impairment caused by fatigue over time. We will pilot the Psychological Outcome Profiles (PSYCHLOPS) tool^34^ to track progress towards reducing the impact of fatigue on the most important functional outcomes for study participants.. As regards confounding variables, anxiety and depression will be screened using the Hospital Anxiety and Depression Scale^35^ while cognitive function will be screened using the Addenbrooke’s Cognitive Examination-III (ACE-III).^36^ We will record prescribed medication at each study time-point. For all these outcomes we will gather pilot data on the magnitude of effect size in the treatment arms versus control, in order to estimate likely effect sizes in a larger trial.

We may in addition pilot instruments to examine the Health Economic costs and potential benefits of these interventions. We will assess health benefits using the EQ-5D which is the nationally-recommended instrument for this specific purpose.^37^

## 4.2 Sampling time-points

T0 (baseline): Routine clinic-demographic information from notes review and GP contact, including past medical and psychiatric history, current medications and doses, and physical functional status. Battery of study materials as outlined. T1 (ten weeks post-randomisation): study materials as outlined. T2 (16 weeks post randomisation): study materials as outlined. In addition, qualitative interviews will be conducted on a subset of participants between 10 and 13 weeks post-randomisation (see Figure 1 and Figure 2 below).

| Activity | SCREENING | T0  BASELINE | Wk 2- 10  INTERVENTION  PHASE | T1 (Wk10)  POST-INTERVENTION | T2 (Wk16)  ENDPOINT |
| --- | --- | --- | --- | --- | --- |
| Brief Fatigue Inventory (BFI) | X |  |  | X | X |
| Patient Written Informed consent | X |  |  |  |  |
| Eligibility Checklist |  | X |  |  |  |
| Medical History |  | X |  |  |  |
| Demographics |  | X |  |  |  |
| Concomitant Medications |  | X |  | X | X |
| FACIT Fatigue Scale |  | X |  | X | X |
| PSYCHLOPS Pre-therapy |  | X |  |  |  |
| PSYCHLOPS Post-therapy |  |  |  | X | X |
| EQ-5D |  | X |  | X | X |
| HADS |  | X |  | X | X |
| ACE-III |  | X |  | X | X |
| Randomisation |  | X |  |  |  |
| Health Coaching Initial Consultation form ^1^ |  |  | X |  |  |
| Health Coaching appointments ^1, 2^ |  |  | X |  |  |
| Health Coaching Activity Log ^1, 3^ |  |  | X |  |  |
| Patient Activation interview ^4^ |  |  | X |  |  |
| Qualitative interview ^1, 5^ |  |  | X |  |  |

***Figure 2:*** *Treatment and Examination Schedule*

1. For patient randomised to Arm 2 or 3 only.
2. Health Coaching will be delivered for eight weeks. Over this period participants will receive a total of up to 8 sessions lasting up to 45 minutes each, by telephone, Skype, or in person (clinic).
3. Participants will record standardised information as often as possible, up to a maximum of each day.
4. For patients randomised to Arm 3 only. PA coach will meet the participant and conduct a standardised semi-structured interview. Participants will be offered a second PA interview, identical in structure to the first, after a further four weeks.
5. Will be conducted in a sub-study of 24 participants within 3 weeks of T1.

## 4.3 Study materials

All participants will complete (at T0, T1 and T2) the measures outlined under ‘**4.1:** **Develop systems and pilot outcome measures’** above. Those receiving Health Coaching will also record data as outlined in the DREEMS model above. Professionals on the study will gather procedural data on recruitment, retention, the technical delivery of Health Coaching, and service use by participants.

## 4.4 Concomitant Therapy

During the study, participants will be under stable follow-up for a primary brain tumour. Consequently all will have completed primary radiotherapy and/or chemotherapy (if necessary). Some may continue on routine medications to manage symptoms such as anti-epileptic drugs. Medications and doses will be recorded observationally for clinico-demographic use. No medication will be prescribed or tested as part of this study.

# 5. SAFETY MONITORING

This is not a trial of a pharmacological compound. We anticipate that the risk of any untoward physical incident as a result of the interventions delivered here is either low, or nil. However brain tumour patients are medically ill, so for completeness and transparency of governance we include the following section. Arguably it is also important because the Health Coaching Intervention is not wholly sedentary: one of the components involves attaining a goal for number of steps taken per day (measured automatically by fitbit-style monitor).

## 5.1 Definitions

**Adverse Event (AE):** An adverse event (AE) is any untoward medical occurrence in a study participant which does not necessarily have a causal relationship with the study treatments or procedures. An adverse event can therefore be any unfavourable and unintended sign (including an abnormal laboratory finding), symptom, or disease temporally associated with a treatment or procedure, whether or not considered related.

**Adverse Reaction (AR):** All noxious and unintended responses related to a study treatment or procedure should be considered adverse reactions.

**Serious Adverse Event (SAE):** Defined as any untoward medical occurrence in a participant that:

1. Results in death;
2. Is life-threatening;
3. Requires hospitalisation or prolongation of existing hospitalisation;
4. Results in persistent or significant disability or incapacity, or;
5. Is otherwise considered medically significant by the Investigator

Important medical events may also be considered serious if they jeopardise the subject or require an intervention to prevent one of the above consequences.

The term “life-threatening” refers to an event in which the participant was at risk of death at the time of the event; it does not refer to an event which hypothetically might have caused death if it were more severe.

Hospitalisations planned prior to enrolment in the trial or for social reasons will not be considered as SAEs. Treatment in an A&E department of less than 24 hours or on an out-patient basis that does not meet any other serious criteria should not be considered as an SAE.

## 5.2 Expected events

This section lists those events that are to be regarded as expected for reporting purposes. Note that all are common or potentially common events in patients with a primary brain tumour under normal circumstances.

- Fatigue due to tumour or tumour progression
- Epileptic seizures in a patient with epilepsy, or due to tumour progression
- Functional physical deterioration due to tumour or tumour progression
- Psychological distress due to tumour or tumour progression
- Worsening cognitive impairment due to tumour, tumour progression, or late-delayed radiation reaction
- Tumour progression per se
- Death (due to a complication of tumour)
- Late-delayed radiation reaction
- Pulmonary embolus
- Falls and injury secondary to falls

## 5.3 Recording of Adverse Events

All adverse events occurring after signing of informed consent through to 30 days after final study procedure will be recorded in the subject’s notes and transcribed to the CRF.

Any medical conditions or diseases present prior to signing of informed consent should only be considered an adverse event if there is a worsening of the condition.

## 5.4 Recording and Reporting of Serious Adverse Events

**Contact Details for Reporting SAEs**

SCTRU Fax: +44 131 275 7512

SCTRU Email: NSS.SCTRU@nhs.net

SCTRU Telephone: +44 131 275 7276/ 4278 (Mon – Fri 9am-4pm)

All serious adverse events that occur after the signing of written informed consent and within 30 days after the final study procedure will be recorded on the SAE report form. In addition, any SAE that occurs more than 30 after final study procedure and is deemed to be related to a study procedure should be recorded on the SAE report form. The SAE report form must be signed by the Principal Investigator of the centre involved and faxed to SCTRU within 24 hours of the Investigator first becoming aware of the event. All initial SAE reports should contain the following minimum information:

- Reporter details
- At least one suspect procedure
- At least one subject identifier (trial number/patient initials)
- Adverse Event term
- Causality assessment

A fax or email receipt will be sent to the relevant centre by SCTRU to acknowledge receipt of the SAE report form, and SCTRU will notify the Chief Investigator (CI). The sponsor will be notified of all SAE’s which occur in the form of a line listing which will be sent to them by SCTRU every 2 months.

Any SAE that has been assessed as related will be forwarded to the CI by SCTRU. Any SAE that is deemed to be both **related (**ie resulted from administration of any of the research procedures) and **unexpected (**ie not listed in the protocol as an expected occurrence) will be notified to the Research Ethics Committees within 15 days of the CI becoming aware of the event

Related and unexpected SAE’s should be reported to the REC using the ‘Non-CTIMP safety report to REC form’. This should be signed by the CI and include a statement on the assessment of the implications, if any, for the safety of study participants and how will these be addressed. The coordinator of the REC should acknowledge receipt of the safety report within 30 days. The MHRA do not require to be notified of SAEs within this trial, as the study does not involve the use of an investigational medicinal product. <http://www.hra.nhs.uk/resources/during-and-after-your-study/progress-and-safety-reporting/>. SCTRU will notify the PI’s at all of the participating centres of the occurrence of any related and unexpected SAE’s

There is no requirement to submit annual safety reports to the REC in addition to the information provided through the annual [progress report](https://www.hra.nhs.uk/approvals-amendments/managing-your-approval/progress-reports/).

## 5.5 Pregnancies

Any pregnancy in a trial participant that occurs during study participation should be reported to SCTRU within 24 hours of the site RA or PI becoming aware of its occurrence, using the contact details in Section 5.3. The SCTRU will ensure that the information is passed to the relevant Health Coach to make them aware.

# 6. DATA MANAGEMENT

All data will be handled, computerised and stored in accordance with the Data Protection Act 1998 and NHS National Services Scotland Confidentiality Guidelines.

## 6.1 Data Collection

**Health coaching:**

At the first health coaching session, participants will be given a home diary to record their progress. The health coach will initiate a secure end-to-end [NHS.net](http://NHS.net)-based email thread, by emailing the participant’s preferred email address from an [nhs.net](http://nhs.net) account, with their study code number and “[Secure]” in the subject line. All study email communication between the health coach and the participant will use this encrypted Study Thread. At intervals by agreement, the participant will take a clear picture of their completed home diary on their smartphone. They will access their email account and email the health coach on the secure Study Thread, attaching the picture of their diary. In this way the health coach will gather data securely, with minimal effort for participants and without the need for a third-party app or the posting of hard copies of the diary. Follow-up appointments will be guided by these securely-emailed images of the participant’s home diary. After the appointment the health coach will forward the diary image via [nhs.net](http://nhs.net) to the RA/RN.

Alternatively, participants may choose to attend follow-up in person. In this case they will simply be asked to bring their diary with them to the appointment. The health coach will use the diary to guide the appointment. At the end of the appointment the health coach will scan the diary and save the image file on an NHS Lothian-encrypted memory stick. They will then use [nhs.net](http://nhs.net) to email the image file to the RA/RN . Once acknowledged by the RA/RN, the scanned image file will be deleted from the memory stick.

**Patient activation:**

The Patient Activation interviews will not be audio-recorded. The coaches will not take written notes during the interview. Immediately after the interview, the patient activation coach will write an email, send to the RA/RN via [nhs.net](http://nhs.net), summarising the interview. The RA/RNs will upload the text to the central study database.

**At site:**

Data generated from T0, T1 and T2 interviews will be collected at site, The data will then be checked and validated by SCTRU. The data collected will include:

- initial clinical details at randomisation
- concomitant medications
- adverse events
- survival
- withdrawal
- protocol deviations

## 6.2 Record Keeping and Archiving

Study documentation will be retained at site until the end of follow up. The documentation will then be archived using an NHS-approved service, according to current legislative requirements.

# 7. STATISTICS

## 7.1 Sample Size

60 participants will be randomised to one of three study arms:

- Control (n=20);
- Health Coaching (n=20);
- Health Coaching plus Patient Activation intervention (n=20).

## 7.2 Power considerations

In line with NIHR guidance^25^ this study is neither intended nor powered to study the efficacy of Health Coaching. Rather our sample size of 60 patients (20 patients per group) is based on what is reasonably sufficient to study feasibility. Equally the subsample of 24 patients who will receive a qualitative interview is in line with accepted practice in the qualitative research field.

## 7.3 Exit strategy to a definitive trial

We will develop systems, relationships, and experience in the four centres necessary to running a definitive trial. We will in particular pilot the FACIT-F scale^33^ as a possible primary outcome measure, given that it is a frequently used fatigue scale for which validated minimal clinically important differences have been published. We have additionally conducted a power calculation to illustrate the capacity for a definitive trial. Assuming: (1) a standard 2-arm RCT and 1-1 randomisation schedule; (2) a typical standard deviation in the FACIT-F of SD=12; (3) Alpha (p)= 0.05, then in order to have 90% power to detect a minimal clinically important difference in the FACIT-F of 8 points, we would require n=49 participants per arm. The current feasibility study will inform us further about rates of recruitment and attrition, and likely effect sizes. Given the prevalence of fatigue in patients with a brain tumour, and because we will pilot systems over a wide geographical catchment, we see a viable exit strategy from this feasibility study.

## 7.4 Analysis Plan

Final analysis will be performed at the end of the study, i.e. when all patients have completed their assessments and all data has been cleaned and the database locked.

Statistical quality assurance will be carried out to correct spurious data and to mimimise the level of missing data.

Analysis will be carried out on all patients randomised to the study.

Number of patients recruited over a 12 month period will be tabulated overall by site and treatment arm. This will be compared to the target of 20 patients per arm.

Number and proportion of patients retained within the trial to the T2 endpoint (16 weeks after randomisation) will be tabulated overall and by site and treatment arm. This will be compared to the target that at least 60% of patients will be retained until the T2 endpoint.

The mean change (from baseline to 16 week follow up) in outcome scale scores with 95% confidence intervals will be calculated for each treatment arm.

## 7.5 End of Study

The End of Study will be performed when all patients have completed their assessments at the end of the 16 week follow up and all data has been cleaned and database finalised.

# 8. ACCESS TO SOURCE DATA/ DOCUMENTS

The investigator, by accepting to participate to this protocol, agrees to co-operate fully with any quality assurance visit undertaken by third parties, including representatives from the Sponsor, SCTRU or the Coordinating Centre, or regulatory authorities, as well as to allow direct access to documentation pertaining to the clinical trial (including CRFs, source documents, hospital patient charts and other study files) to these authorised individuals.

# 9. QUALITY CONTROL AND QUALITY ASSURANCE

Quality control will be maintained through adherence to the Principles of ICH GCP (Appendix 3) and the SCTRU or coordinating centre’s SOPs. The coordinating centre will monitor receipt of CRFs and evaluate incoming CRFs for compliance with the protocol, inconsistencies and missing data.

## 9.1 Monitoring Visits

There will be no monitoring visits performed for this study.

## 9.2 Data Monitoring Committee/ Trial Steering Committee

An independently-chaired Data Monitoring Committee (DMC)/ Trial Steering Committee (TSC) will be established to provide overall supervision of the trial, in particular; trial progress, adherence to protocol, patient safety, and consideration of new information. The joint DMC/TSC will meet 6 monthly in the first instance and then 6 monthly thereafter (and at any other time at the committee’s discretion). The committee will receive regular reports from SCTRU. TheDMC/TSC will be jointly chaired by **Prof. Anthony Byrne** (University of Cardiff) and **Prof. Martin Klein** (Vrie Universiteidt Medisch Centrum, Amsterdam), in rotation.

# 10. ETHICAL CONSIDERATIONS

Favourable ethical opinion by a Research Ethics Committee will be obtained before the trial is started. The trial will be carried out according to guidelines of good clinical practice (GCP) as defined by paragraph 28 and Schedule 1 Part 2 of the Medicines for Human Use (Clinical Trials) Regulations, 2004, and the Clinical Trials Directive (2001/20/EC) elsewhere in the European Union and follow the principles of research governance.

## 10.1 Participant Confidentiality

The participant’s full name, date of birth and hospital number will be collected to enable tracing through national records. The personal data recorded on all records will be regarded as confidential, and to preserve anonymity, only the trial number and initials will be recorded on CRFs.

The PI (or delegate) at each site will keep a log of the site’s participants’ trial numbers, names, addresses, email address, phone number(s) and hospital numbers. The PI must ensure that confidentiality is maintained and that all trial documents (e.g. consent forms) are maintained in strict confidence.

SCTRU will maintain the confidentiality of all data and will not reproduce or disclose any information by which participants could be identified. Participants will only be referred to by trial number and initials in any essential trial related correspondence, including Case Report Forms and Serious Adverse Event Reports.

All patient-identifiable data will be handled, computerised and stored in accordance with the Data Protection Act 1998 and NHS National Services Scotland Confidentiality Guidelines.

## 10.2 Informed Consent

All participants will be informed of the aims of the study, the procedures and possible hazards to which they will be exposed, and the mechanism of treatment allocation. They will be informed as to the strict confidentiality of their data, but that their medical records may be reviewed for trial purposes by authorised individuals other than their treating physician. It will be emphasised that the participation is voluntary and that patients are allowed to refuse further participation in the protocol whenever they want. This will not prejudice their subsequent care.

Documented informed consent will be obtained for all participants in the study before they are enrolled. This will be done in accordance with the national and local regulatory requirements and will conform to guidelines on Good Clinical Practice. That is, “the written informed consent form should be signed and personally dated by the patient or by the patient’s legally acceptable representative”.

Copies of the Patient Information Sheets and consent forms are provided in the supporting materials accompanying this protocol.

All Patient Information Sheets & Informed Consent Forms will be version controlled and dated and this information will always be stated in any communication with ethics committees.

# 11. RESEARCH GOVERNANCE

**Sponsor (NHS Lothian)** – NHS Lothian will act as study sponsor, with co-sponsorship from The University of Edinburgh . The sponsors will have overall responsibility for the design, co-ordination and management of the study. These include:

- Trial authorisation including responsibility for the protocol and obtaining approvals
- Ensuring that the trial is conducted according to GCP guidelines (22,23)
- Review of SAEs

**Clinical Trials Unit** – The sponsor has delegated the responsibility for overall project management, data management and monitoring to Scottish Clinical Trials Research Unit, NHS National Services Scotland. Responsibilities include:

- 1. Assistance with completion of the IRAS form and REC communication
  2. Production of trial specific documentation (i.e. CRFs)
  3. Facilitating set up of trial centres
  4. Data management
  5. Monitoring
  6. Safety Monitoring

Central study co-ordination, data collection, monitoring and organisation of the data for the statistical analyses will be undertaken by the Scottish Clinical Trials Research Unit, NHS National Services Scotland, which has processes in place to ensure that the study will not open to recruitment until appropriate favourable opinions have been obtained from an independent research ethics committee, and NHS Research and Development departments (R&D).

**Statistical Analysis** – A Principal Information Analyst (Robert Hill), based at SCTRU, Edinburgh will undertake the final analysis arising for this study.

**Local Project Teams** – These will consist of a consultant Surgeon, Oncologist, Neurologist, or Clinical Nurse Specialist (responsible for introducing the patient to the study and partially ensuring eligibility), and a Research Assistant (responsible for co-ordination of all aspects of data collection). Centres are specifically responsible for conducting the trial in accordance with the protocol, Standard Operating Procedures (SOPs), the trial agreement and Good Clinical Practice.

**Trial Steering Committee/Data MonitoringCommittee** –. The TSC/DMC, including members of the research team, a statistician, and lay representation, will be responsible for the progress and conduct of the study. Specific issues that will be looked at include: recruitment, retention, tolerability of Health Coaching, withdrawals of consent, schedule reductions, and adverse events. A Trial Management Group (TMG) will meet monthly..

# 12. FINANCING AND INSURANCE

This study is wholly funded by The Brain Tumour Charity. Indemnity for participating hospitals is provided by the usual NHS indemnity arrangements.

# 13. PUBLICATION POLICY

All presentations and publications relating to the trial will be authorised by the Trial Management Group. The main trial results will be published in the name of the trial in a peer-reviewed journal, on behalf of all Collaborators. The manuscript will be prepared by the Trial Management Group, representatives from SCTRU, NHS National Services Scotland, and high accruing clinicians. The trials offices and all participating Centres and clinicians will be acknowledged in this publication. Any data that might detrimentally affect the progress of the trial will not be released prior to the end of the trial. No investigator may present or attempt to publish data concerning participants, which is directly relevant to the questions posed in the trial, until the main results have been published.

# 14. REFERENCE LIST

1. Cancer Research UK. [http://www.cancerresearchuk.org/health-professional/cancer-statistics/statistics-by-cancer-type/brain-tumours/incidence#heading-Zero](http://www.cancerresearchuk.org/health-professional/cancer-statistics/statistics-by-cancer-type/brain-tumours/incidence%25252523heading-Zero) Accessed 25^th^ Oct 2016.

2. Struik K, Klein M, Heimans JJ, Gielissen MF, Bleijenberg G, Taphoorn MJ, Reijneveld JC, Postma TJ. Fatigue in low-grade glioma. J Neurooncol 2009; 92:73-8.

3. Valko PO, Siddique A, Linsenmeier C, Zaugg K, Held U, Hofer S. Prevalence and predictors of fatigue in glioblastoma: a prospective study. Neuro Oncology 2015; 17:274-281.

4. The Brain Tumour Charity. Losing myself: the reality of life with a brain tumour. 2015; The Brain Tumour Charity.

5. Armstrong TS, Vera-Bolanos E, Acquaye AA, Gilbert MR, Ladha H, Mendoza T. The symptom burden of primary brain tumors: evidence for a core set of tumor and treatment-related symptoms. Neuro Oncol. 2015 Aug 19. pii: nov166.

6. Boele FW, Klein M, Reijneveld JC, Verdonck-de Leeuw IM, Heimans JJ. Symptom management and quality of life in glioma patients. CNS Oncol. 2014; 3:37-47.

7. Bower JE. Cancer-related fatigue—mechanisms, risk factors, and treatments. Nat Rev Clin Oncol 2014; 11:597-609.

8. Bower JE. Treating cancer-related fatigue: the search for interventions that target those most in need. Journal of Clinical Oncology 2012; 30:4449-4450.

9. Cramp F, Byron-Daniel J. Exercise for the management of cancer-related fatigue in adults. Cochrane Database of Systematic Reviews 2012, Issue 11. Art. No.: CD006145. DOI: 10.1002/14651858.CD006145.pub3.

10. Goedendorp MM, Gielissen MFM, Verhagen CAHHVM, Bleijenberg G. Psychosocial interventions for reducing fatigue during cancer treatment in adults. Cochrane Database of Systematic Reviews 2009, Issue 1. Art. No.: CD006953. DOI: 10.1002/14651858.CD006953.pub2.

11. Gielissen MF, Verhagen S, Witjes F: Effects of cognitive behaviour therapy in severely fatigued disease-free cancer patients compared with patients waiting for cognitive behavior therapy: A randomized controlled trial. J Clin Oncol 2006; 24:4882-4887.

12. Bower JE, Garet D, Sternlieb B, et al: Yoga for persistent fatigue in breast cancer survivors: A randomized controlled trial. Cancer 2011; 118:3766-3755.

13. <http://bio-mechanix.co.uk> Website of our collaborator Garry Anderson, who has structured a lifestyle intervention into the manner we adopt for study here. Accessed 24th Oct 2016

14. Willems RA, Bolman CA, Mesters I, Kanera IM, Beaulen AA, Lechner L. Short-term effectiveness of a web-based tailored intervention for cancer survivors on quality of life, anxiety, depression, and fatigue: randomized controlled trial. Psychooncology 2016; doi: 10.1002/pon.4113.

15. Hibbard JH, Stockard J, Mahoney ER, Tusler M. Development of the Patient Activation Measure (PAM): conceptualizing and measuring activation in patients and consumers. Health Serv Res 2004; 39:1005-1026.

16. Greene J, Hibbard JH. Why does patient activation matter? An examination of the relationships between patient activation and health-related outcomes. J Gen Intern Med 2012; 27(5): 520-526.

17. Day J, Yust-Katz S, Cachia D, Wefel J, Katz LH, Tremont I, Bulbeck H, Armstrong T, Rooney AG. Interventions for the management of fatigue in adults with a primary brain tumour. Cochrane Database of Systematic Reviews 2016, Apr 13;4:CD011376. doi: 10.1002/14651858.CD011376.pub2.

18. Boele FW, Douwe L, de Groot M, van Thuijl HF, Cleijne W, Heimans JJ, Taphoorn MJ, Reijneveld JC, Klein M. The effect of modafinil on fatigue, cognitive functioning, and mood in primary brain tumor patients: a multicenter randomized controlled trial. Neuro-Oncology 2013; 15:1420-1428.

19. Page BR, Shaw EG, Lu L, Bryant D, Grisell D, Lesser GJ, Monitto DC, Naughton MJ, Rapp SR, Savona SR, Shah S, Case D, Chan MD. Phase II double-blind placebo-controlled randomized study of armodafinil for brain radiation-induced fatigue. Neuro-Oncology 2015; 17:1393-1401.

20. Butler JM Jr, Case LD, Atkins J, Frizzell B, Sanders G, Griffin P, Lesser G, McMullen K, McQuellon R, Naughton M, Rapp S, Steiber V, Shaw EG. A phase III, double-blind, placebo-controlled prospective randomized clinical trial of d-threo-methylphenidate HCl in brain tumor patients receiving radiation therapy, International Journal of Radiation Oncology, Biology, Physics 2007; 69:1496-1501.

21. Gehring K, Sitskoorn MM, Gundy CM, Sikkes SAM, Klein M, Postma TJ, van den Bent MJ, Beute GN, Enting RH, Kappelle AC, Boogerd W, Veninga T, Twijnstra A, Boerman DH, Taphoorn MJB, Aaronson NK. Cognitive rehabilitation in patients with gliomas: a Randomized, Controlled Trial. Journal of Clinical Oncology 2009; 27:3712-3722.

22. Locke DEC, Cerhan JH, Wu W, Malec JF, Clark MM, Rummans TA, Brown PD. Cognitive rehabilitation and problem-solving to improve quality of life of patients with primary brain tumours: a pilot study. Journal of Supportive Oncology 2008; 6:383-391.

23. MacDonald L, JLA Neuro-Oncology Priority Setting Partnership. Top 10 priorities for clinical research in primary brain and spinal cord tumours: final report of the James Lind Alliance Priority Setting Partnership in Neuro-Oncology. JLA Neuro-Oncology Priority Setting Partnership, 2015. Also <http://www.neuro-oncology.org.uk/priorities/index.php>

24. Craig P, Dieppe P, Macintyre S, Michie S, Nazareth I, Petticrew M. Developing and evaluating complex interventions: the new Medical Research Council guidance. BMJ 2008;337:a1655.

25. [http://www.rds-sw.nihr.ac.uk/dloads/RfPB_Feasibility_Trials_Guidance.pdf](http://www.rds-sw.nihr.ac.uk/dloads/rfpb_feasibility_trials_guidance.pdf) Accessed 1st August 2017.

26. Hibbard J, Mahoney E, Stockard J, Tusler M. Development and testing of a short form of the patient activation measure. Health Serv Res 2005; 40:1918-1930.

27. Prey JE, Qian M, Restaino S, Hibbard J, Bakken S, Schnall R, Rothenberg G, Vawdrey DK, Masterton Creber R. Reliability and validity of the patient activation measure in hospitalized patients. Patient Educ Couns 2016; pii: S0738-3991(16)30294-4. doi: 10.1016/j.pec.2016.06.029.

28. Dilts R. Changing Belief Systems with NLP. Meta Publications, Cupertino, California, 1990.

29. Bandura A. Self-Efficacy: Toward a unifying theory of behavioral change. Psychological Review 1977; 84:191–215.

30. Lorig K, Holman H. Self-management education: History, definition, outcomes, and mechanisms. Ann Behav Med. 2003; 26:1–7.

31. Ritchie R, Spencer E. Qualitative data analysis for applied policy research. In: Bryman A, Burgess R, editors. *Analyzing Qualitative Data*. London: Routledge, 1994:173-94.

32. Mendoza TR, Wang XS, Cleeland CS, Morrisey M, Johnson BA, Wendt JK, Huber SL. The rapid assessment of fatigue severity in cancer patients: use of the Brief Fatigue Inventory. Cancer 1999; 85(5):1186-96.

33. Cella D, Lai JS, Stone A. Self-reported fatigue: one dimension or more? Lessons from the Functional Assessment of Chronic Illness Therapy - Fatigue (FACIT-F) questionnaire. Support Care Cancer 2011;19(9):1441-1450.

34. Ashworth M, Evans C, Clement S. Measuring psychological outcomes after cognitive behaviour therapy in primary care: a comparison between a new patient-generated measure “PSYCHLOPS” (Psychological Outcome Profiles) and “HADS” (Hospital Anxiety and Depression Scale). Journal of Mental Health 2009; 18:169-77. See also <http://www.psychlops.org.uk/about.html>

35. Zigmond AS, Snaith RP. The Hospital Anxiety and Depression scale. Acta Psychiatrica Scandinavia 1983; 67:361-370.

36. Hsieh S, Schubert S, Hoon C, Mioshi E, Hodges JR. Validation of the Addenbrooke's Cognitive Examination III in frontotemporal dementia and Alzheimer's disease. Dement Geriatr Cogn Disord 2013; 36:242-250.

37. EuroQol. About EQ-5D. <http://www.euroqol.org/about-eq-5d.html> Accessed 12th Oct 2015.

**Additional references**

The Medicines for Human Use (Clinical Trials) Regulations 2004 (Statutory Instrument 2004:1031).

The Medicines for Human Use (Clinical Trials) Amendment Regulations 2006 (Statutory Instrument 2006:1928).

Directive 2001/20/EC of the European Parliament and of the Council of 4 April 2001 on the approximation of the laws, regulations and administrative provisions of the member states relating to the implementation of good clinical practice in the conduct of clinical trials on medicinal products for human use.

Commission Directive 2005/28/EC of 8 April 2005 laying down principles and detailed guidelines for good clinical practice as regards investigational medicinal products for human use, as well as the requirements for authorisation of the manufacturing or importation of such products.

Integrated Addendum to ICH E6 (R1): Guideline for Good Clinical Practice E6(R2); Current step 5 adopted by CHMP 15 Dec 2016

ICH Harmonised Tripartite Guideline for Good Clinical Practice (ICH E6) 1996.

NHS National Services Scotland Confidentiality Guidelines (version 1.1.1a); 2012.

### **Appendix 1a – Investigator Statement (SCTRU Copy)**

BT-LIFE

Brain Tumours, Lifestyle Interventions, and Fatigue Evaluation

Principal Investigator Declaration

I acknowledge receipt of version <#> date <dd/mmm/yyyy> of the BT-LIFE trial protocol (REC favourable ethical opinion received <dd/mmm/yyyy>) and I agree to perform this trial in accordance with this version of the protocol and Good Clinical Practice.

I understand that the safety of the patient is my first concern.

Print Name: --------------------------------------------------------

Hospital: --------------------------------------------------------

Signed: --------------------------------------------------------

Date: --------------------------------------------------------

Please return this copy to: BT-LIFE Trial Coordinator

Scottish Clinical Trials Research Unit,

Gyle Square,

1 South Gyle Crescent,

Edinburgh,

EH12-9EB

### **Appendix 1b – Investigator Statement (Investigator Copy)**

BT-LIFE

Brain Tumours, Lifestyle Interventions, and Fatigue Evaluation

Principal Investigator Declaration

I acknowledge receipt of version <#> date <dd/mmm/yyyy> of the BT-LIFE trial protocol (REC favourable ethical opinion received <dd/mmm/yyyy>) and I agree to perform this trial in accordance with this version of the protocol and Good Clinical Practice.

I understand that the safety of the patient is my first concern.

Print Name: --------------------------------------------------------

Hospital: --------------------------------------------------------

Signed: --------------------------------------------------------

Date: --------------------------------------------------------

Please retain this copy and file in Investigator Site File

### **Appendix 1c - The Principles of ICH Good Clinical Practice**

1. Clinical trials should be conducted in accordance with the ethical principles that have their origin in the Declaration of Helsinki, and that are consistent with GCP and the applicable regulatory requirement(s).
2. Before a trial is initiated, foreseeable risks and inconveniences should be weighed against the anticipated benefit for the individual trial subject and society. A trial should be initiated and continued only if the anticipated benefits justify the risks.
3. The rights, safety, and well-being of the trial subjects are the most important considerations and should prevail over interests of science and society.
4. The available nonclinical and clinical information on an investigational product should be adequate to support the proposed clinical trial.
5. Clinical trials should be scientifically sound, and described in a clear, detailed protocol.
6. A trial should be conducted in compliance with the protocol that has received prior institutional review board (IRB)/independent ethics committee (IEC) approval/ favourable opinion.
7. The medical care given to, and medical decisions made on behalf of, subjects should always be the responsibility of a qualified physician or, when appropriate, of a qualified dentist.
8. Each individual involved in conducting a trial should be qualified by education, training, and experience to perform his or her respective task(s).
9. Freely given informed consent should be obtained from every subject prior to clinical trial participation.
10. All clinical trial information should be recorded, handled, and stored in a way that allows its accurate reporting, interpretation and verification. This principle applies to all records referenced in this guideline, irrespective of the type of media used.
11. The confidentiality of records that could identify subjects should be protected, respecting the privacy and confidentiality rules in accordance with the applicable regulatory requirement(s).
12. Investigational products should be manufactured, handled, and stored in accordance with applicable good manufacturing practice (GMP). They should be used in accordance with the approved protocol.
13. Systems with procedures that assure the quality of every aspect of the trial should be implemented. Aspects of the trial that are essential to ensure human subject protection and reliability of trial results should be the focus of such systems.

### **Appendix 1d - Schedule for Health Coaching intervention**

1. **Following informed consent and before the first Health Coaching appointment**

The Research Assistant/Research Nurse willcontact the Health Coach to notify them that a patient has been randomized and to schedule the first appointment.

1. **At the first Health Coaching appointment (face to face in clinic)**

The participant will be given a diary to record data by hand.

Participants will be asked to start recording lifestyle information

A Health Coaching ‘Consultation Form’ will be given to all participants to complete.

Discuss Consultation Form

Initial assessment – measure % body fat, blood pressure, body mass index / perform basic biomechanics screening to establish physical ability.

Introduce muscle activation exercises.

Set goal(s) for week ahead.

Participant and Health Coach will organise the next follow up appointment and type (face to face, Skype, phone).

1. **Between appointments**

Participants to record lifestyle information, using the home diary. Information should be recorded as often as possible but it is not essential that it is recorded every day, if this is difficult.

Participants will receive regular reports on progress from their Health Coach.

The Health Coach will record manageability data in the Health Coach Manageability Log.

1. **Follow up appointments (in clinic/home/phone/skype)**

The Health Coach will review recorded lifestyle information recorded since the previous appointment.

New goal/s will be set as appropriate. Participants will be encouraged to maintain goals already in place from the previous appointments.

Participants will be able to ask questions and concerns. These will be recorded by the Health Coach to inform our understanding of the acceptability of the intervention.

**APPENDIX: Lifestyle Information recorded during Health Coaching**

Participants will record standardised lifestyle information daily into a home diary ,

Participants will record lifestyle information on Drink, Rest, Eating, Exercise, Movement and Stress (DREEMS). The recording of each behavioural component of DREEMS is explained below.

**Lifestyle Information (DREEMS)**

**D**rink**:** The number of each daily fluid item (water, milk, juice, tea, coffee and alcohol) will be self-reported daily in the Nudge app or the custom design form.

**R**est: Total sleep time (hours per day) will be self-reported in the Nudge app or the Custom Design Form.

**E**ating**:** Food items will be self-reported in the app or Custom design form. Food items measured include Protein, Fruit, Vegetables, Dairy, Legumes, Nuts, Healthy Fats, Starches and Indulgent food items. These food items can be tailored suit to the participant’s diet.

**E**xercise: Frequency and duration of exercise sessions will be recorded in the app or the Custom Design Form.

**M**ovement**:** Total number of steps taken per day will be recorded objectively by a fitbit-style monitor. The data retrieved will be synced to the app, or written into the custom design form by hand.

**S**tress: Stress will be self-reported using a simple three-stage scale; low/neutral/high. This data will be reported in the app or the custom design form.

### **Appendix 1e - Schedule for Patient Activation intervention**

**Following informed consent and allocation to Group 3**

- The recruiting Research Assistant/Research Nurse will seek the participant’s preference of location of Patient Activation: at home, Skype, telephone, or facetime.
- The Research Assistant/Research Nurse will liaise with *brainstrust* and the participant to arrange the time of the first session. This will be scheduled to occur after the first Health Coaching session.

**First Patient Activation session**

1. The Patient Activation coach (“the coach”) will meet with the participant as arranged, and discuss the aims and use of PA in the context of fatigue and the study.
2. The coach will survey the participant’s current level of Activation by administering the PAM-13 Patient Activation measure. This will be by physical copy for face-to-face coaching, or verbal transcription for distance coaching.
3. Discuss the PAM-13 results and use them to guide the intervention.
4. The coach will lead the participant through the semi-structured “Dilts’ Logical Levels” schedule (see Appendices), focusing on increasing the participant’s skills, knowledge and confidence to manage their own fatigue.
5. Agree suitable goal(s) for the participant to aim for.
6. Arrange next coaching session date and location preference.

**Between sessions**

- The Coach will make notes of their session content and store these, anonymised, on an encrypted NHS memory stick.
- Participants receiving Patient Activation will not be asked to keep a diary of any specifications with respect to PA, between sessions.
- If they choose, they will be able to contact the PA coach to discuss questions between sessions. The PA coach will keep an anonymised record of any such participant-initiated contact using an encrypted NHS memory stick.

**Second Patient Activation session (c. 4 weeks after first)**

1. General discussion of progress since last session.
2. Administer the PAM-13 and discuss any changes between coaching sessions one and two.
3. Apply the FRAME (Feedback, Responsibility, Advice, Menu, and Efficacy) approach to enhance patient self-efficacy in managing their fatigue.
4. Elicit any problems using G.R.O.W. (Goal, Reality, Options, Way forward), to explore options and articulate ways forward.

**APPENDIX: Supporting material for Patient Activation intervention**

**Patient Activation Measure (PAM-13) questions and underlying rationale (used in sessions one and two)**

1. When all is said and done, I am the person who is responsible for managing my health condition.
2. Taking an active role in my own health care is the most important factor in determining my health and ability to function.
3. I am confident that I can take actions that will help prevent or minimise some symptoms or problems associated with my health condition.
4. I know what each of my prescribed medications do.
5. I am confident that I can tell when I need to get medical care, and when I can handle a health problem myself.
6. I am confident I can tell my health care provider concerns I have, even when he or she does not ask.
7. I am confident that I can follow through on medical treatments I need to do at home.
8. I understand the nature and causes of my health condition(s).
9. I know the different medical treatment options available for my health condition.
10. I have been able to maintain the lifestyle changes for my health that I have made.
11. I know how to prevent further problems with my health condition.
12. I am confident I can figure out solutions when new situations or problems arise with my health condition.
13. I am confident that I can maintain lifestyle changes like diet and exercise even during times of stress.

*Lower levels of Patient Activation may indicate:*

- A lower level of knowledge and skills in managing fatigue / health coaching;
- Participant may benefit from taking smaller steps;
- Participant may not understand causes of fatigue or how lifestyle changes may work;
- Participant may not have confidence in their ability to complete health coaching;
- A menu of options, empathetic reflections, and empowering affirmations may benefit.

*Higher levels of Patient Activation may indicate:*

- A higher level of knowledge, skills, and confidence;
- Participant may or may not resent someone telling them what to do;
- Participant may have strong opinions about treatment options and may have already researched ways to improve fatigue;
- Participant may be used to success – potential cause of frustration if progress is not as hoped;
- Supporting their autonomy may benefit.
- Participants with lower PA may benefit from greater direction from a coach, while those with higher PA may require less direction and benefit from a greater focus on autonomy. Therefore, measuring Patient Activation will guide how the coach approaches each session.

**Dilts’ Logical Levels framework and exemplars (used in session one)**

**Purpose**

What do you think health coaching aims to achieve?

Do you think it will have an effect on your fatigue?

What is your hope/desire to achieve by taking part in this project?

**Identity**

What do you see as your role in health coaching?

Do you consider yourself to be a person who is interested in their health?

**Values**

How important is it to you to complete health coaching to your best ability?

Do you believe that health coaching will help your fatigue?

Do you feel that your health coach is important in achieving your goals?

**Capabilities**

How confident are you in achieving the goals and tasks set out by your health coach?

What will you need to complete health coaching to your best ability?

What bits of HC are you looking forward to tackling the most/least?

**Behaviours**

Can you think of things you might do to help you complete health coaching?

Are there any current activities that might not be productive in completing health coaching?

Has the health coach suggested any activities that you’ve already thought about doing?

**Environment**

Who or what in your environment do you think will be important in achieving your goals?

Is there anything in your environment that you find particularly exhausting?

Can you think of things that help reinvigorate you?

**F.R.A.M.E. framework and exemplars (used in session two)**

**F**eedback

Discuss the lowest rated items on the PAM-13. *E.g.* If the participant registered a low score on Item 9, the coach might open a discussion of the perceived importance *to the participant* of knowing about different treatment options.

**R**esponsibility

Assess the participant’s perceived personal responsibility in improving knowledge/confidence. *E.g.* If they do not perceive themselves to have responsibility, the coach might seek to increase their level of Patient Activation by asking the participant about the pros and cons of their current approach.

**A**dvice

Collaborative goal-setting in relation to the lowest PAM-13 score(s).

**M**enu

Discuss the menu of options for self-management relating to issues discussed in *Feedback*

section:

- Start with what the participant feels comfortable aiming for in the short term.

- Move on, if suitable, to longer-term goals.

**E**fficacy

Enhancement strategies for self-efficacy:

- Use previous successes to motivate new advances in their health.

- Break goals into attainable steps

- How can they take the initial steps to achieve this?

- What support will they need?

- Who can help them?

**G.R.O.W. framework and exemplars (used in session two)**

**G**oal

Discuss the goal from first session and any progress in achieving it.

**R**eality

What has happened in achieving the goal?

Have they got any closer in certain aspects – has anything been put back?

Is this goal still realistic?

What has stepped in the way of the goal?

**O**ptions

What has changed/developed that we can apply to achieving the goal?

Are there any recent developments?

Brainstorm possible options to achieving goals.

What is still a limiting factor in your recovery/ability to achieve your goals?

What if this constraint was removed?

**W**ay forward

What is still driving you to make these changes?

What will you do now?

When would you like to do it?

What might set you back

How would you tackle this?
