# Supplementary material for: Lifestyle coaching is feasible in fatigued brain tumor patients: A phase I/feasibility, multi-center, mixed-methods randomized controlled trial
Source: Neurooncol Pract. 2022 Oct 14;10(3):249–60. doi: 10.1093/nop/npac086 (PMC10180387; doi:10.1093/nop/npac086)
Supplement: npac086_suppl_Supplementary_Data_S2 [file npac086_suppl_supplementary_data_s2.docx]

**BT-LIFE Supplementary Results.**

BFI 2 way mixed ANOVA

There were no outliers in the data, as assessed by inspection of a boxplot. Data for all arms at all time points were normally distributed (Shapiro-Wilk’s test p>0.05) except for the HC arm at T2 (p=0.034), with homogeneity of variances (Levene's test *p* > 0.05) and homogeneity of covariances (Box's test *p* = 0.329). The assumption of sphericity was violated for the two-way interaction (Mauchley’s χ^2^[2] = 10.58, *p* = 0.008) for which the Greenhouse-Geisser correction was applied. There was a statistically significant interaction between arm and time on mean BFI score (*F*[3.04, 44.12] = 3.87, *p* =0.015, partial η^2^ = 0.211, ε = 0.761).

No statistically significant differences were observed in mean BFI score between arms at baseline or at T2. However there was a simple main effect of group at T1 *F*(2, 32) = 3.39, *p* =0.046, partial η^2^ = 0.175, with mean BFI score statistically and clinically significantly lower in the HC arm (-2.3 points [95%CI -4.5 to -0.1], *p* = .041) compared to control at this time-point. In addition there was a simple main effect of time lowering BFI in all three arms (Control *F*(2, 22) = 7.06, *p*=.004, partial η^2^ =0.391; HC F(2,20)=10.66, p=0.001, partial η^2^=0.516; HC/AC F(2,16)=12.08, p=0.001, partial η^2^=0.602.)

FACIT-F 2 way mixed ANOVA

There were no outliers in the data as assessed by inspection of a boxplot. Data for all arms at all time points were normally distributed (Shapiro-Wilk’s test p>0.05), with homogeneity of variances (Levene's test *p* > 0.05) and homogeneity of covariances (Box's test *p* = 0.342). The assumption of sphericity was met for the two-way interaction (Mauchley’s χ^2^[2] = 1.24, *p* = 0.538). There was a statistically significant interaction between arm and time on FACIT-F score (*F*[4, 58] = 4.74, *p* =0.002, partial η^2^=0.276).

No statistically significant differences were observed in FACIT-F score between arms at baseline. Estimated marginal means suggested an improvement in FACIT-F scores in both intervention arms, but variance was high and main effects of group at T1 and T2 were not statistically significant. However there was a simple main effect of time improving FACIT-F scores in the combined arm (HC/AC F=[2,18]=11.58, p=0.001, partial η^2^=0.563; 95%CI= 2.3 to 23.3 [T1] and 3.5 to 21.0 [T2] point improvement versus control).

Calculation of sample size for a future trial.

Assuming an effect size of -1 (in line with the effect sizes observed in this trial), α=0.05, β=0.20, and observing a correlation of 0.64 between T0 (baseline) and T1 BFI scores with a retention rate of 74%, n=46 patients would be required per arm in a future definitive trial (**Fig 4D**).
