# Supplementary figures and images for: Lifestyle coaching is feasible in fatigued brain tumor patients: A phase I/feasibility, multi-center, mixed-methods randomized controlled trial
Source: Neurooncol Pract. 2022 Oct 14;10(3):249–60. doi: 10.1093/nop/npac086 (PMC10180387; doi:10.1093/nop/npac086)

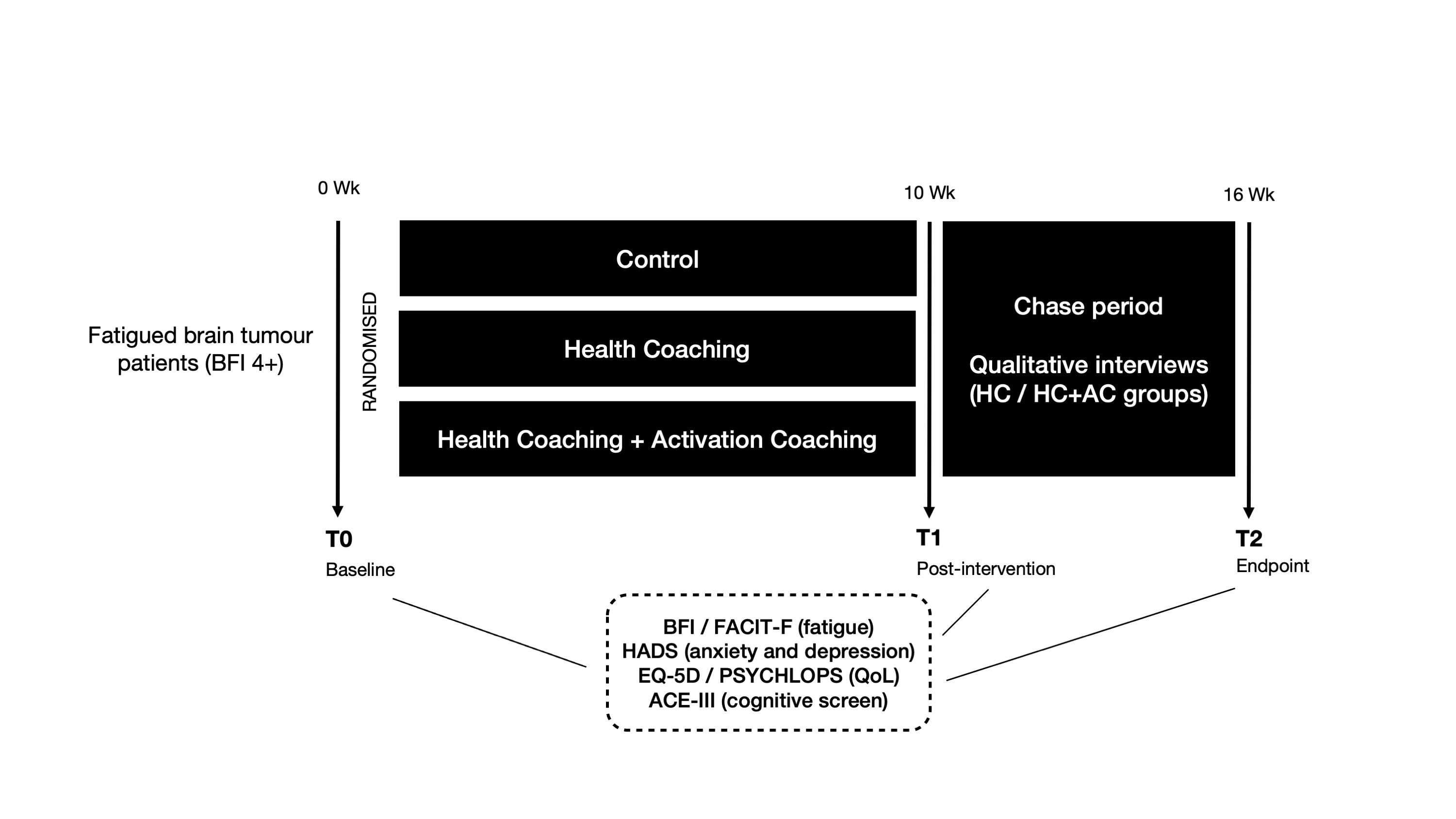

Supplement: npac086_suppl_Supplementary_Figure_S1 [file npac086_suppl_supplementary_figure_s1.jpeg]

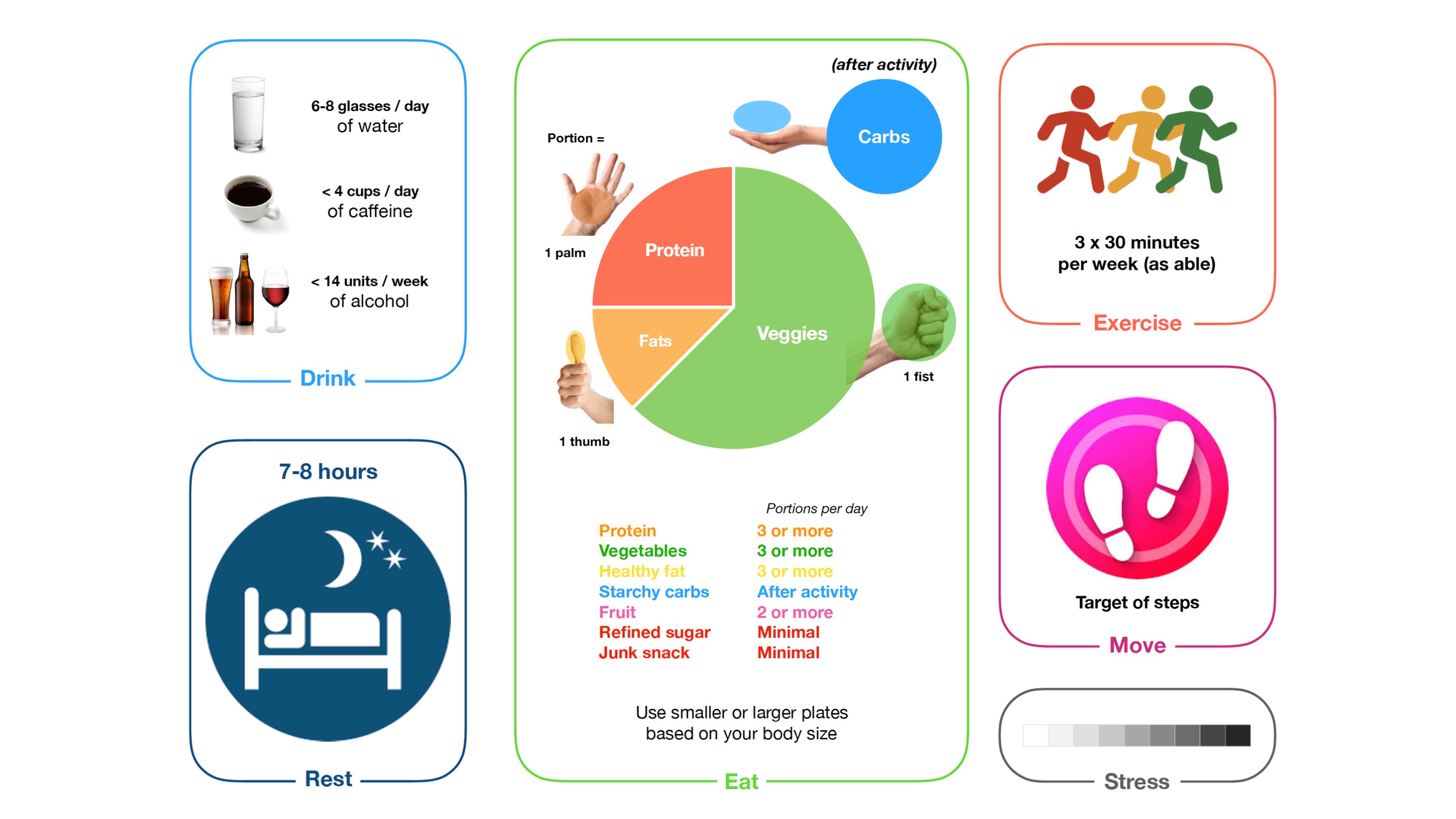

Supplement: npac086_suppl_Supplementary_Figure_S2 [file npac086_suppl_supplementary_figure_s2.jpeg]

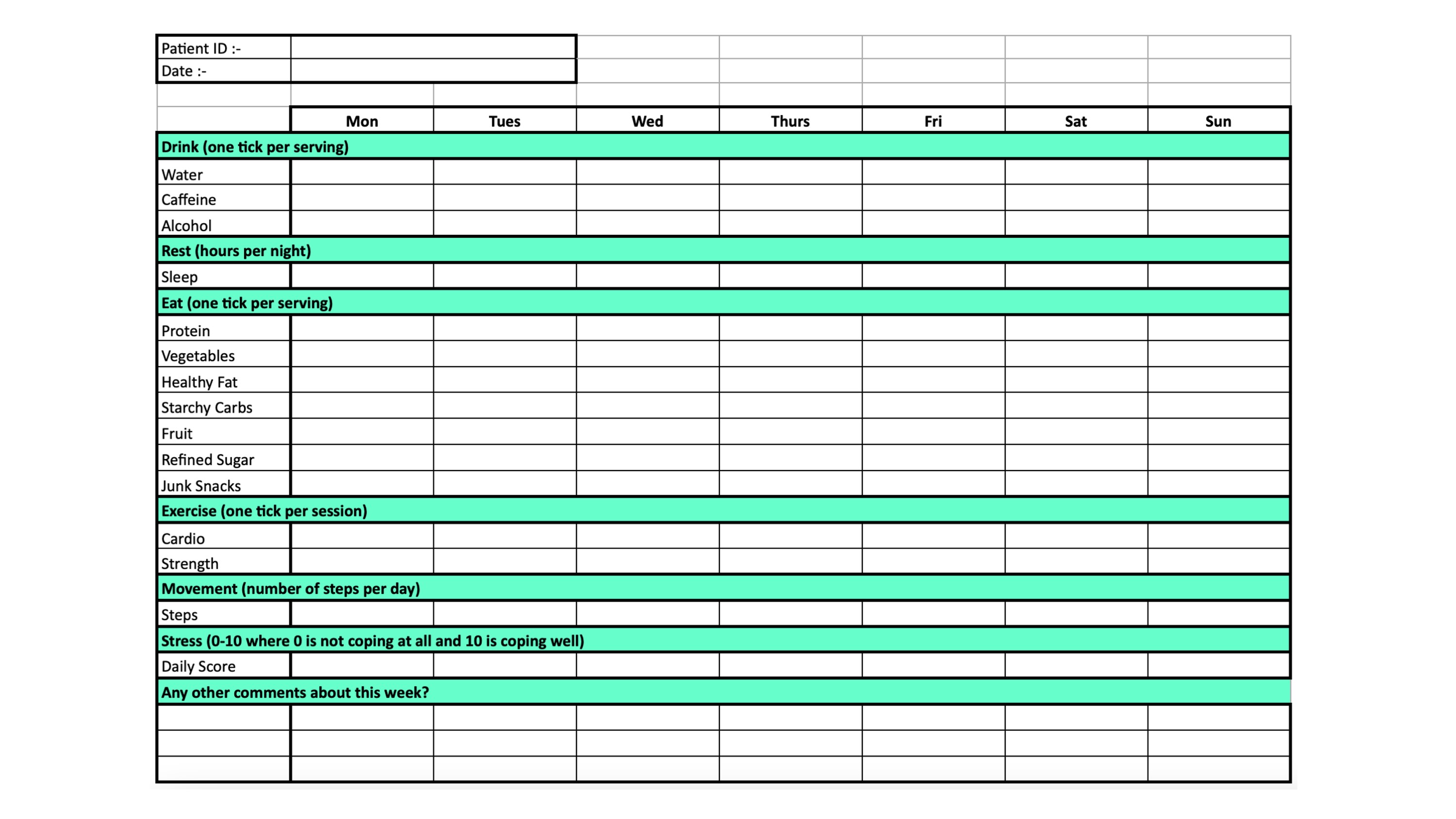

Supplement: npac086_suppl_Supplementary_Figure_S3 [file npac086_suppl_supplementary_figure_s3.jpeg]

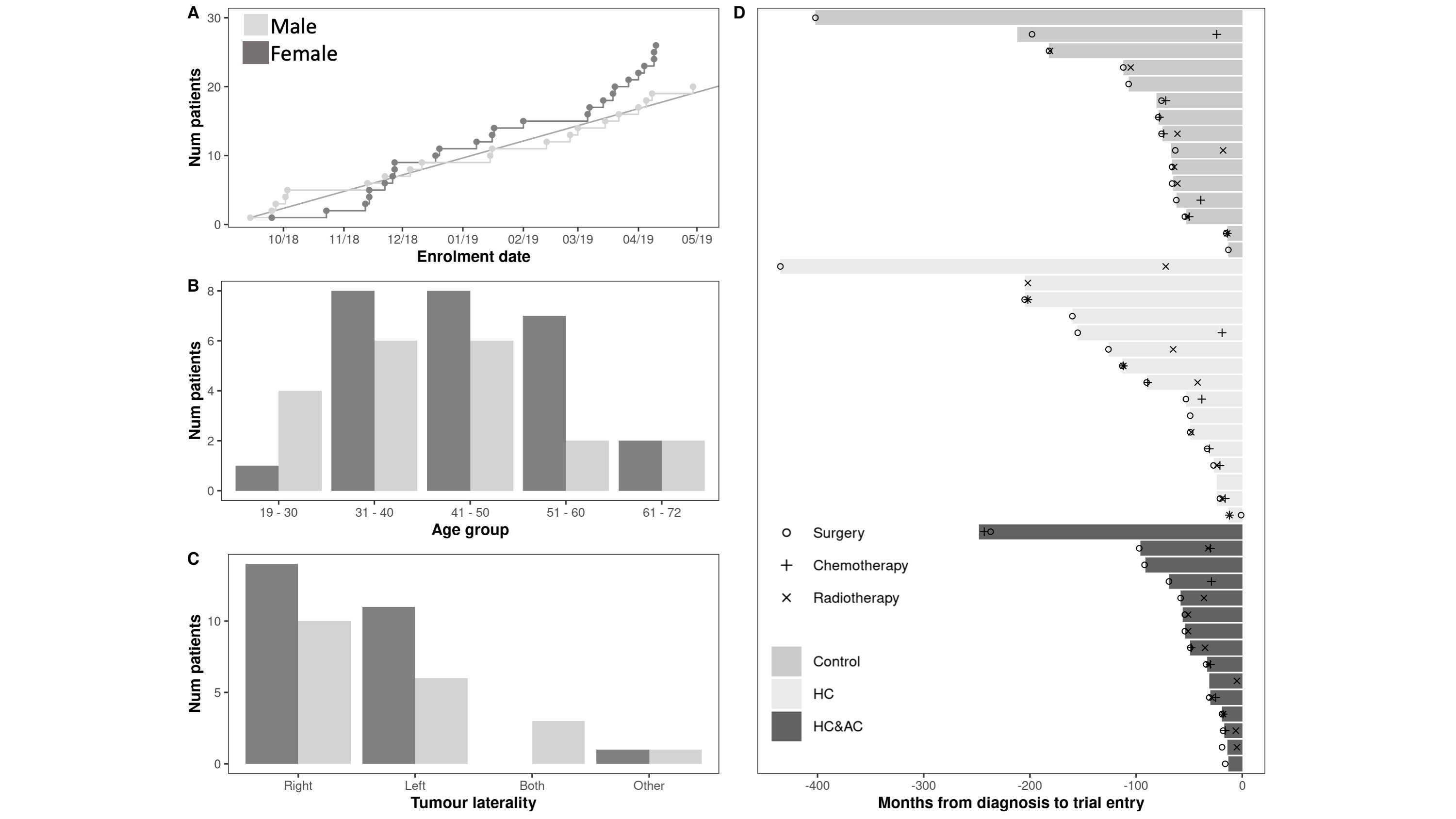

Supplement: npac086_suppl_Supplementary_Figure_S4 [file npac086_suppl_supplementary_figure_s4.jpeg]

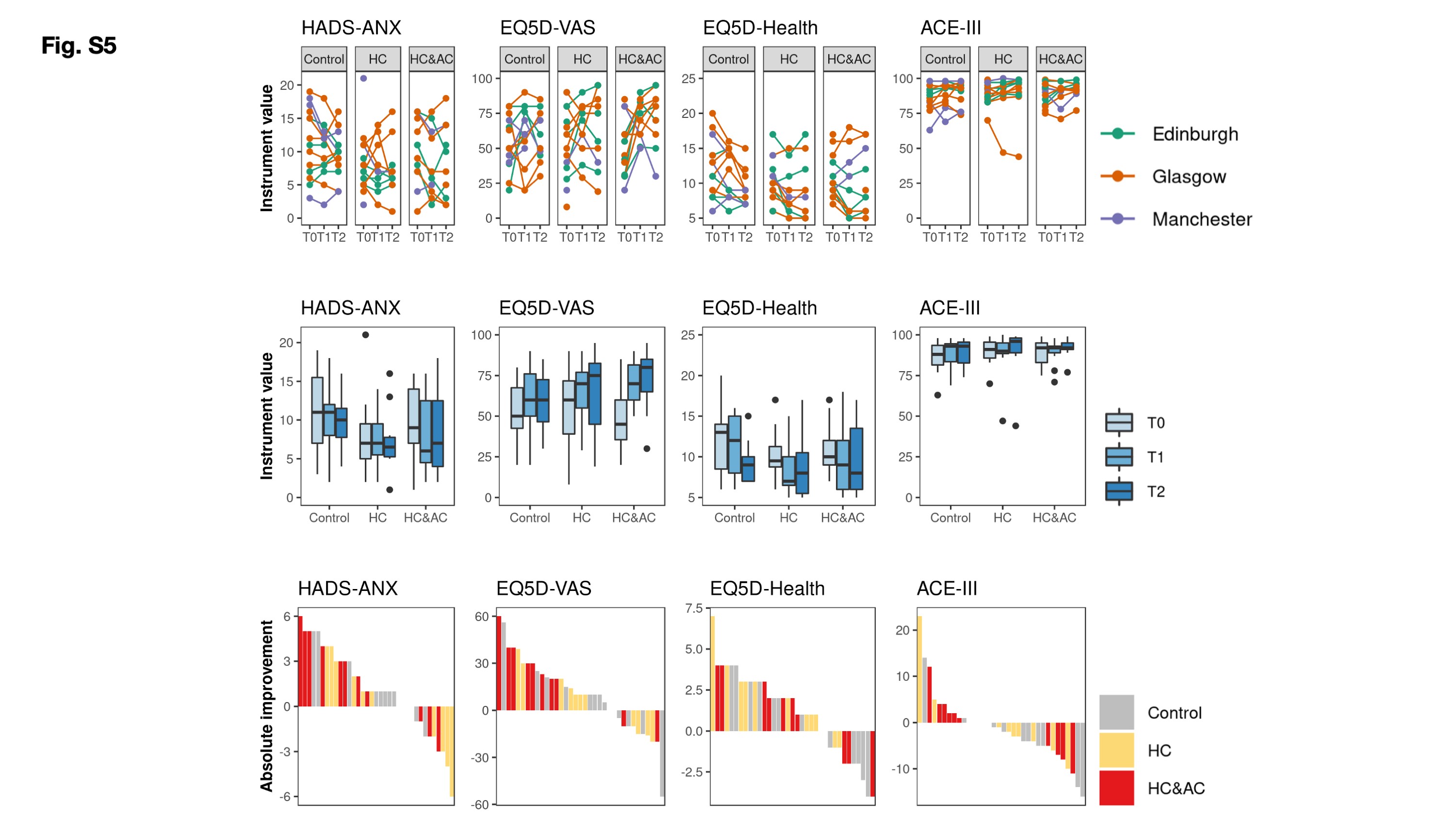

Supplement: npac086_suppl_Supplementary_Figure_S5 [file npac086_suppl_supplementary_figure_s5.jpeg]
