# Supplementary material for: Lifestyle coaching is feasible in fatigued brain tumor patients: A phase I/feasibility, multi-center, mixed-methods randomized controlled trial
Source: Neurooncol Pract. 2022 Oct 14;10(3):249–60. doi: 10.1093/nop/npac086 (PMC10180387; doi:10.1093/nop/npac086)
Supplement: npac086_suppl_Supplementary_Table_S1 [file npac086_suppl_supplementary_table_s1.docx]

| Table S1 | | | | | | |
| --- | --- | --- | --- | --- | --- | --- |
|  | **Site** | **Arm** | **N** | **Date of first** | **Date of last** | **%** |
| **RECRUITMENT** |  |  |  |  |  |  |
|  | Edinburgh | Control | 4 | 2018-10-02 | 2019-03-06 | - |
|  | Edinburgh | Health Coaching | 5 | 2018-09-27 | 2019-04-01 | - |
|  | Edinburgh | Health + Activation Coaching | 5 | 2018-09-14 | 2019-03-27 | - |
|  | Glasgow | Control | 8 | 2018-10-03 | 2019-04-09 | - |
|  | Glasgow | Health Coaching | 8 | 2018-09-25 | 2019-04-05 | - |
|  | Glasgow | Health + Activation Coaching | 8 | 2018-09-25 | 2019-04-04 | - |
|  | Manchester | Control | 3 | 2018-11-22 | 2019-04-10 | - |
|  | Manchester | Health Coaching | 3 | 2018-11-22 | 2019-04-29 | - |
|  | Manchester | Health + Activation Coaching | 2 | 2018-12-20 | 2019-03-14 | - |
|  | ALL | ANY | 46 | 2018-09-14 | 2019-04-29 | - |
|  |  |  |  |  |  |  |
| **RETENTION** | Edinburgh | Control | 3 | - | - | 75 |
|  | Edinburgh | Health Coaching | 4 | - | - | 80 |
|  | Edinburgh | Health + Activation Coaching | 4 | - | - | 80 |
|  | Glasgow | Control | 6 | - | - | 75 |
|  | Glasgow | Health Coaching | 6 | - | - | 75 |
|  | Glasgow | Health + Activation Coaching | 6 | - | - | 75 |
|  | Manchester | Control | 3 | - | - | 100 |
|  | Manchester | Health Coaching | 1 | - | - | 33 |
|  | Manchester | Health + Activation Coaching | 1 | - | - | 50 |
|  | ALL | ANY | 34 | - | - | 73.9 |
