# Supplementary material for: Lifestyle coaching is feasible in fatigued brain tumor patients: A phase I/feasibility, multi-center, mixed-methods randomized controlled trial
Source: Neurooncol Pract. 2022 Oct 14;10(3):249–60. doi: 10.1093/nop/npac086 (PMC10180387; doi:10.1093/nop/npac086)
Supplement: npac086_suppl_Supplementary_Table_S5 [file npac086_suppl_supplementary_table_s5.docx]

| **Model term** | **Estimate** | **lower 95%** | **upper 95%** | **SE** | **p value** | **R sq** | **Adjusted R sq** |
| --- | --- | --- | --- | --- | --- | --- | --- |
| β0: Intervention | 2.495 | 1.795 | 3.195 | 0.344 | 0.000 | 0.286 | 0.263 |
| β1: Control offset | -1.988 | -0.382 | 1.397 | 0.556 | 0.001 |  |  |
|  |  |  |  |  |  |  |  |
| β0: Intervention intercept | 4.450 | 2.643 | 6.257 | 0.885 | 0.000 | 0.425 | 0.368 |
| β1: Control offset | -5.332 | -8.285 | -2.380 | 1.446 | 0.001 |  |  |
| β2: HADS-D T0 slope (Intervention) | -0.249 | -0.463 | -0.034 | 0.105 | 0.025 |  |  |
| β3: HADS-D T0 slope difference (Control) | 0.419 | 0.076 | 0.762 | 0.168 | 0.204 |  |  |
